# Titanium dioxide and carbon black nanoparticles disrupt neuronal homeostasis via excessive activation of cellular prion protein signaling

**DOI:** 10.1186/s12989-022-00490-x

**Published:** 2022-07-15

**Authors:** Luiz W. Ribeiro, Mathéa Pietri, Hector Ardila-Osorio, Anne Baudry, François Boudet-Devaud, Chloé Bizingre, Zaira E. Arellano-Anaya, Anne-Marie Haeberlé, Nicolas Gadot, Sonja Boland, Stéphanie Devineau, Yannick Bailly, Odile Kellermann, Anna Bencsik, Benoit Schneider

**Affiliations:** 1 INSERM, UMR-S 1124, 75006 Paris, France; 2 UMR-S 1124, Université Paris Cité, 75006 Paris, France; 3 Institut Des Neurosciences Cellulaires Et Intégratives, CNRS UPR 3212, Université de Strasbourg, 67084 Strasbourg, France; 4 Plateforme Anatomopathologie Recherche, INSERM 1052, CNRS 5286, Centre Léon Bérard, Centre de Recherche en Cancérologie de Lyon (CRCL), Université Claude Bernard Lyon 1, Université de Lyon, 69373 Lyon, France; 5 CNRS UMR 8251, Unité de Biologie Fonctionnelle Et Adaptative, Université Paris Cité, 75013 Paris, France; 6 ANSES Laboratoire de Lyon, French Agency for Food, Environmental and Occupational Health & Safety (ANSES), Université Claude Bernard Lyon 1, 69364 Lyon, France

**Keywords:** Nanoparticles, PrP^C^ receptor, Signaling, TNFα receptors, Aβ peptides, Neuroinflammation, Nanoneurotoxicity, Alzheimer’s disease

## Abstract

**Background:**

Epidemiological emerging evidence shows that human exposure to some nanosized materials present in the environment would contribute to the onset and/or progression of Alzheimer’s disease (AD). The cellular and molecular mechanisms whereby nanoparticles would exert some adverse effects towards neurons and take part in AD pathology are nevertheless unknown.

**Results:**

Here, we provide the prime evidence that titanium dioxide (TiO_2_) and carbon black (CB) nanoparticles (NPs) bind the cellular form of the prion protein (PrP^C^), a plasma membrane protein well known for its implication in prion diseases and prion-like diseases, such as AD. The interaction between TiO_2_- or CB-NPs and PrP^C^ at the surface of neuronal cells grown in culture corrupts PrP^C^ signaling function. This triggers PrP^C^-dependent activation of NADPH oxidase and subsequent production of reactive oxygen species (ROS) that alters redox equilibrium. Through PrP^C^ interaction, NPs also promote the activation of 3-phosphoinositide-dependent kinase 1 (PDK1), which in turn provokes the internalization of the neuroprotective TACE α-secretase. This diverts TACE cleavage activity away from (i) TNFα receptors (TNFR), whose accumulation at the plasma membrane augments the vulnerability of NP-exposed neuronal cells to TNFα -associated inflammation, and (ii) the amyloid precursor protein APP, leading to overproduction of neurotoxic amyloid Aβ40/42 peptides. The silencing of PrP^C^ or the pharmacological inhibition of PDK1 protects neuronal cells from TiO_2_- and CB-NPs effects regarding ROS production, TNFα hypersensitivity, and Aβ rise. Finally, we show that dysregulation of the PrP^C^-PDK1-TACE pathway likely occurs in the brain of mice injected with TiO_2_-NPs by the intra-cerebro-ventricular route as we monitor a rise of TNFR at the cell surface of several groups of neurons located in distinct brain areas.

**Conclusion:**

Our in vitro and in vivo study thus posits for the first time normal cellular prion protein PrP^C^ as being a neuronal receptor of TiO_2_- and CB-NPs and identifies PrP^C^-coupled signaling pathways by which those nanoparticles alter redox equilibrium, augment the intrinsic sensitivity of neurons to neuroinflammation, and provoke a rise of Aβ peptides. By identifying signaling cascades dysregulated by TiO_2_- and CB-NPs in neurons, our data shed light on how human exposure to some NPs might be related to AD.

**Supplementary Information:**

The online version contains supplementary material available at 10.1186/s12989-022-00490-x.

## Background

The growing incidence of neurodegenerative diseases worldwide is suspected to relate to increased human exposure to diverse pollutants. This includes some nanosized materials, the so-called nanoparticles (NPs), which display the capacity to cross easily physiological barriers and to highly react with biological systems (for review, see [1] and references therein). Incriminated NPs are the ultrafine particulate matter present in the air, but also manufactured NPs, such as silver, amorphous silica, titanium dioxide (TiO_2_), and carbon black (CB) nanoparticles, for which the amount put on the market augments each year, and that concern both workers and consumers ([2], and for review, see [3] and references therein). In regards to the huge diversity of NP applications in everyday products (e.g., food, cosmetics, pigment industry, etc.) and the release of NPs by those products, humans are regularly exposed to these nanosized particles by inhalation, ingestion, and/or cutaneous routes. Chronic exposure to NPs, even at a low dose, would increase the frequency of NPs translocation to the brain, thus raising the possibility of an involvement of NPs in brain disorders, including neurodegenerative diseases (for review, see [1] and references therein).

According to the Organization for Economic Cooperation and Development, Alzheimer’s disease (AD) is the most common dementia in humans whose burden increases since 1990 and currently concerns 50 million individuals worldwide [4]. This neurodegenerative disease slowly destroys memory and thinking skills due to the accumulation and deposition in the brain of neurotoxic amyloid Aβ peptides as senile plaques. Aβ peptides originate from the proteolytic processing of the amyloid precursor protein (APP) by the amyloidogenic β- and γ-secretases [5]. In AD subjects, there is an overproduction of Aβ peptides that would depend on several events acting more or less in parallel, *i.e.*, an increase of APP expression and the amyloidogenic APP processing [6, 7], reduction of the protective APP cleavage by α-secretases [8, 9], and/or impairment of Aβ-degrading enzymes [10]. For instance, epidemiological and experimental studies document that human exposure to environmental chemical pollutants such as pesticides or metals is associated with an increased risk of dementia and *idiopathic* AD later in life (for review, see [11] and references therein, [12–14]). This notably relates to the capacity of these chemicals to increase Aβ levels, promote aggregation and fibrils of Aβ42, and disrupt Aβ clearance [14]. More recently, airborne nanoparticulate matter was also incriminated in AD as young individuals living in air-polluted Metropolitan Mexico City exhibited AD pathological signs in their brainstems [15]. The complex composition of the ambient ultrafine particulate matter (aerodynamic diameter < 100 nm) however renders difficult the analysis of the mechanisms by which airborne nanoparticles would affect the nervous system. Because airborne nanoparticles are primarily made of carbonaceous material, engineered carbon black nanoparticles (CB-NPs) are sometimes used as a surrogate of airborne nanoparticles [16]. Such engineered CB-NPs display a more homogenous and well-defined physicochemical composition than airborne nanoparticles and a comparable morphology. This implies that engineered NPs would also spur AD onset and/or contribute to AD progression in the context of occupational or environmental human exposure [1].

Among the most produced engineered NPs (all external dimensions < 100 nm) are the carbon black (CB-NPs) and titanium dioxide (TiO_2_-NPs) nanoparticles [17]. Manufactured CB-NPs are widely used in rubber and as black pigment. Despite an in vivo harmful action of inhaled CB-NPs in mice, the impact of CB-NPs on brain functions and the neurotoxicity of CB-NPs remain poorly characterized and deserve more investigation. It is reported that a single exposure of adult mice to pure CB-NPs does not modify the level of IL-1β pro-inflammatory cytokine in the central nervous system (CNS) right after the administration of the nanoparticles [18]. When pregnant mice are exposed by the airway to CB-NPs, neurotoxicological signs are induced in the offspring, including reactive astrogliosis [19] and an increase of β-sheet-rich proteins around blood vessels [20]. As concerns nanosized TiO_2_ particles, TiO_2_-NPs are metallic oxide nanoparticles widely used in food and cosmetic industries, as a modifier/enhancer of white pigment, UV-blocker, or anti-microbial agent thanks to their photocatalytic activity. As for other metallic NPs that can reach the central nervous system in mice and humans via the olfactory and/or trigeminal nerves, as well as the systemic circulation [21–23], TiO_2_-NPs can cross the blood–brain barrier and accumulate in the brain [24, 25], notably in the hippocampus [26], a brain structure involved in memory processes and affected early and severely in AD [27]. In the hippocampus, different forms of TiO_2_-NPs were shown to exert some adverse effects such as oxidative stress, mitochondrial dysfunction, gliosis, and to provoke neuronal lesions [1, 28–30]. Exploiting reconstructed neuronal networks, TiO_2_-NPs were also reported to severely impair the electrical activity of neurons [31]. However, the cellular and molecular mechanisms by which CB- and TiO_2_-NPs affect neuronal homeostasis and place neurons on the path to degenerate are unknown.

The cellular prion protein (PrP^C^) is well known for its implication not only in prion diseases (for review, see [32] and references therein) but also Alzheimer’s disease [9, 30, 33]. PrP^C^ is an ubiquitous protein mostly expressed in neurons, tethered to the outer leaflet of the plasma membrane via a GlycosylPhosphatidylInositol (GPI) moiety, that distributes in detergent-resistant microdomains, *i.e.* lipid-rafts or caveolae [34]. With the help of the 1C11 neuronal stem cell line that differentiates into serotonergic (1C11^5−HT^) or noradrenergic (1C11^NE^) neurons upon appropriate induction [35], we assigned a signaling function to GPI-anchored PrP^C^, which acts as a cell surface receptor or co-receptor [36] and controls diverse signaling effectors [37]. This includes the NADPH oxidase and the subsequent nontoxic production of ROS that contribute to the activation of redox-sensitive targets [38, 39]. We further evidenced that PrP^C^ interaction with the amyloidogenic prion peptide 106–126 corrupts PrP^C^ signaling in neuronal cells with the overstimulation of NADPH oxidase and the onset of oxidative stress conditions [40]. More recently, we showed that the interaction of PrP^C^ with neurotoxic prions (PrP^Sc^) or Aβ peptides and subsequent dysregulation of PrP^C^ signaling cancel the neuroprotective cleavage activity of TACE α-secretase (ADAM17) at the plasma membrane of prion-infected or Alzheimer’s neurons, respectively [9, 41]. This dysregulation of TACE renders diseased neurons highly sensitive to TNFα-associated inflammation and amplifies the production of PrP^Sc^ in prion diseases and Aβ peptides in AD but also in prion diseases [9, 42]. Because PrP^C^ binds oligomers, aggregates, and/or fibrils of PrP^Sc^ in prion diseases [43], and oligomers of Aβ peptides in AD [33, 44, 45], the cellular prion protein is suspected to act more generally as a broad sensor for diverse aggregates of amyloid proteins [46]. Of note, the physicochemical properties of many NPs give them the ability to form aggregates/agglomerates in biological fluids [17]. Focusing on TiO_2_- and CB-NPs, the present study investigates whether PrP^C^ would also be a neuronal cell surface receptor for aggregates/agglomerates of those nanoparticles. The interaction between TiO_2_- or CB-NPs and PrP^C^ would corrupt PrP^C^-coupled signaling effectors, leading to neuronal cell dysfunction and the induction of molecular signs of Alzheimer’s disease.

## Results

### TiO_2_ and CB nanoparticles bind recombinant cellular prion protein PrP^C^

**Fig. 1:**
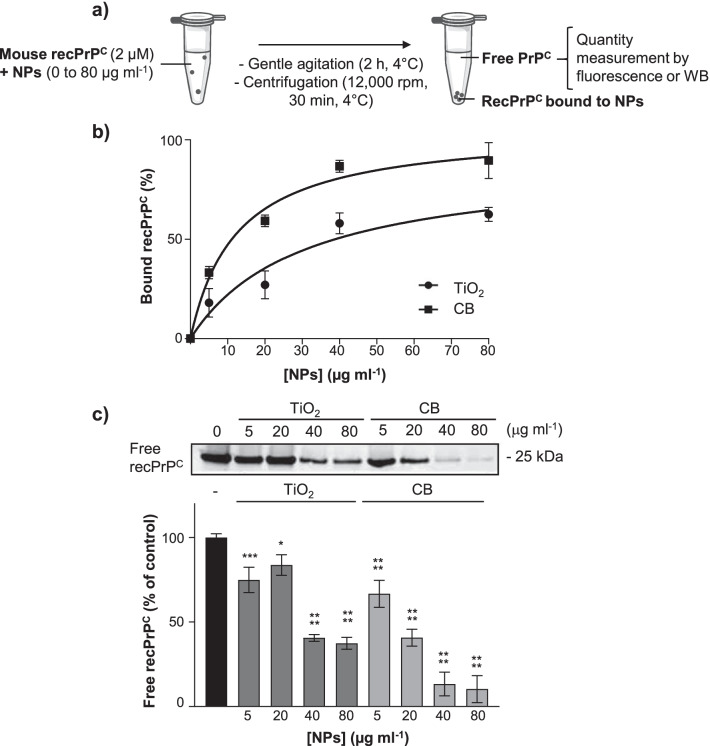
TiO_2_ and CB nanoparticles interact with PrP^C^. **a** Schematic of the experimental procedure to assess the interaction between full-length recombinant mouse PrP^C^ (recPrP^C^, 2 µM) and TiO_2_- or CB-NPs (0–80 µg ml^−1^) exploiting the intrinsic fluorescence of PrP^C^ or by western blotting (WB). **b** Fluorescence titration curves showing the binding of TiO_2_- or CB-NPs to full-length mouse recPrP^C^. The quantity of recPrP^C^ bound with nanoparticles was deduced by subtracting the fluorescence level of titrated free PrP^C^ to the fluorescence level of total PrP^C^ measured in the absence of nanoparticles. Fitting hyperbolic curves were calculated with the help of the Kaleidagraph Software (Abelbeck Software). **c** Representative Western-blot and quantification histogram showing decrease of free recPrP^C^ amount in the supernatant of the centrifuged reaction medium between recPrP^C^ and TiO_2_- or CB-NPs. The experiments were performed three times in triplicates. Values are means ± SEM. *denotes *p* < 0.05, ***p < 0.001, ****p < 0.0001 versus recPrP^C^ incubated without nanoparticles

To first assess whether TiO_2_- (P25) or CB- (FW2) NPs would interact with cellular prion protein PrP^C^, we performed in vitro binding assays using commercial full-length recombinant mouse PrP folded into PrP^C^. PrP^C^ (2 µM) was incubated at 4 °C for 2 h with increasing concentrations of TiO_2_- or CB-NPs (0–80 µg ml^−1^) (Additional file 1: Table S1) under gentle agitation. The mix was then centrifuged at 13,523 g for 30 min and residual PrP^C^ free in the supernatant was titrated exploiting the intrinsic fluorescence of PrP^C^ (λ_exc_ = 280 nm; λ_em_ = 340 nm). The quantity of PrP^C^ bound with nanoparticles was deduced by subtracting the fluorescence level of titrated free PrP^C^ to the fluorescence level of PrP^C^ (2 µM) measured in the absence of nanoparticles (Fig. 1a). Figure 1b shows that both TiO_2_- and CB-NPs interact with recombinant full-length PrP^C^. The quantity of PrP^C^ bound with nanoparticles varied with the nanoparticle concentration according to a hyperbolic curve compatible with the equilibrium of interaction NP + PrP^C^ = PrP^C^(NP). Hyperbolic fitting of the titration data indicated an affinity for PrP^C^ of CB (K_D_ of 12.4 ± 2.2 µg ml^−1^) higher than that measured with TiO_2_ (K_D_ of 30.4 ± 10.9 µg ml^−1^).

To exclude that free nanoparticles would have quenched PrP^C^ fluorescence leading to an overestimation of the quantity of nanoparticles bound to PrP^C^, we also measured by Western blotting the level of recombinant free PrP^C^ present in the supernatant after centrifugation. Quantification of PrP^C^ level confirmed that TiO_2_- and CB-NPs both interact with the protein with an affinity stronger for CB than for TiO_2_ (Fig. 1c).

These in vitro data demonstrate that TiO_2_ and CB nanoparticles, two NPs with distinct chemical composition and physicochemical properties [17, 47], both bind PrP^C^.

### PrP^C^ facilitates binding of TiO_2_ and CB nanoparticles to the plasma membrane of 1C11 precursor and serotonergic 1C11^5−HT^ neuronal cells

**Fig. 2:**
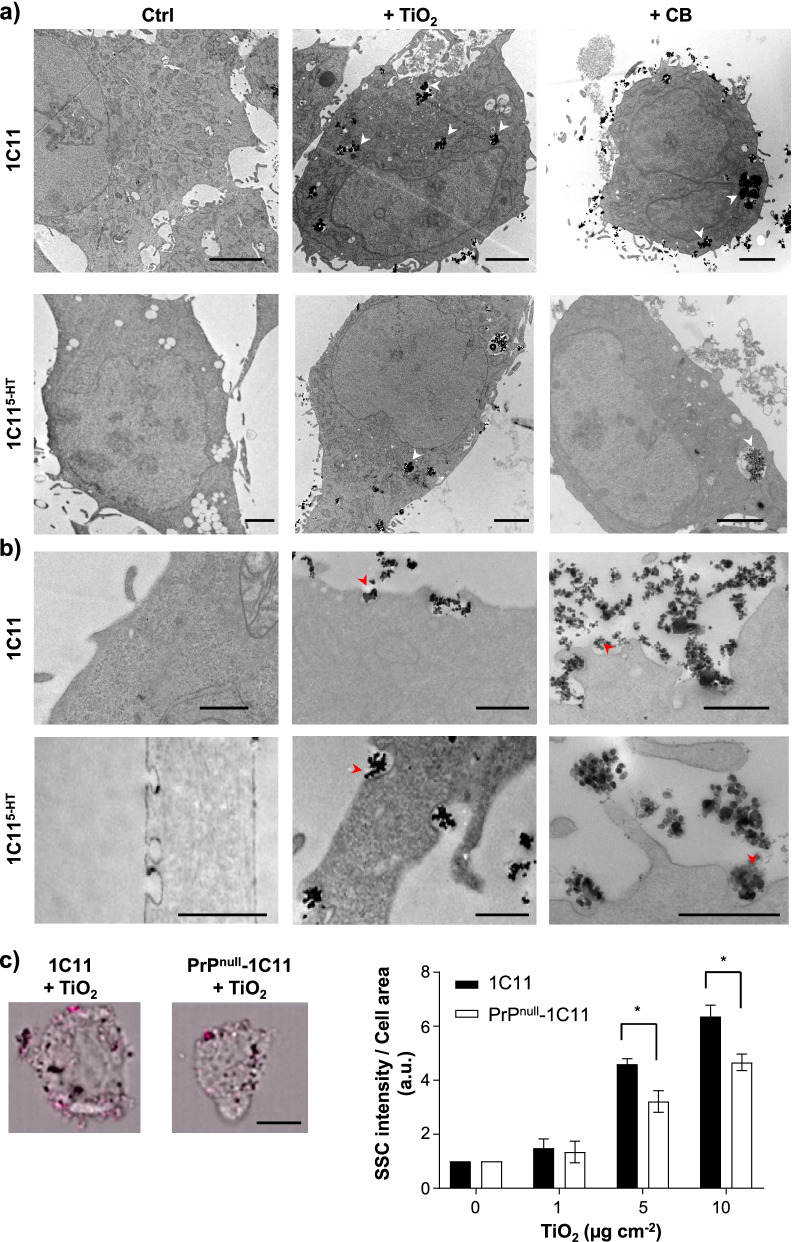
PrP^C^ facilitates nanoparticle interaction with plasma membrane of 1C11 precursors and 1C11^5−HT^ neuronal cells. **a**, **b** Transmission Electron Microscopy experiments showing large aggregates of TiO_2_- and CB-NPs (white arrows) within 1C11 precursor cells and 1C11^5−HT^ neuronal cells **a** and small aggregates of TiO_2_- and CB-NPs (red arrows) at the plasma membrane of 1C11 and 1C11^5−HT^ cells **b** after 24 h exposure to 10 µg cm^−2^ nanoparticles. Scale bar = 2 µm in **a**. Scale bar = 0.5 µm in **b**. **c** Light scattering-based FACS analysis of interacting TiO_2_-NPs with the surface of 1C11 and PrP^null^-1C11 cells exposed up to 10 µg cm^−2^ nanoparticles. (Left) Representative bright-field images of 1C11 and PrP^null^-1C11 cells exposed to 5 µg cm^−2^ TiO_2_-NPs merged with the light scattering signal of TiO_2_-NPs (pink signal) adsorbed to the plasma membrane after internal masking of cells (see Methods). (Right) Quantification histogram of TiO_2_-NPs present at the surface of 1C11 and PrP^null^-1C11 cells. Scale bar = 5 µm. The experiments were performed three times in triplicates. Values are means ± SEM. *denotes *p* < 0.05 versus 1C11 cells exposed to nanoparticles

To next probe whether TiO_2_- and CB-NPs would interact with PrP^C^ present at the cell surface, we exploited the 1C11 neuroectodermal cell line, a neuronal stem cell endowed with the capacity to differentiate into serotonergic 1C11^5−HT^ neuronal cells upon appropriate induction [48]. Whatever the differentiation state, 1C11 and 1C11^5−HT^ cells endogenously express PrP^C^ at the cell surface at similar levels [49]. In the first set of experiments, 1C11 and 1C11^5−HT^ cells were exposed to TiO_2_- or CB-NPs (10 µg cm^−2^) for 24 h, and cell distribution of nanoparticles was analyzed by transmission electron microscopy (TEM). For both types of nanoparticles, TEM analyses revealed the presence of large aggregates of TiO_2_- and CB-NPs within 1C11 and 1C11^5−HT^ cells, likely reflecting endocytosis of nanoparticles (Fig. 2a). Small aggregates of TiO_2_- and CB-NPs were also found attached to the plasma membrane of 1C11 and 1C11^5−HT^ cells in domains that resembled clathrin-coated pits and coated caveolae (Fig. 2b), where PrP^C^ notably distributes [34, 48].

To explore whether PrP^C^ would contribute to the interaction of nanoparticles with the plasma membrane of 1C11 cells, we took advantage of both the light scattering properties of TiO_2_-NPs and the PrP^null^-1C11 cells that are 1C11 cells chronically repressed for PrP^C^ expression by at least 95% [50]. To avoid endocytosis of the nanoparticles by cells, PrP^null^-1C11 cells and their PrP^C^ expressing counterparts were exposed to increasing concentrations of TiO_2_-NPs (0–10 µg cm^−2^) for 15 min at 4 °C in PBS plus azide (0.05%). TiO_2_-NPs adsorbed at the plasma membrane were then titrated by flow cytometry [51]. Because of the low sensitivity of the method, a significant interaction of TiO_2_-NPs with PrP^C^-expressing 1C11 cells started to be detected for TiO_2_-NP concentrations higher than 1 µg cm^−2^. The quantity of TiO_2_-NPs attached to the cell surface of 1C11 cells increases with the TiO_2_-NP exposure concentration (Fig. 2c). Of note, the formation of large aggregates of TiO_2_-NPs at concentrations upper than 10 µg cm^−2^ made probing the nanoparticle-cell interaction by flow cytometry impossible. Due to this technical limitation, TiO_2_-NP binding at the surface of 1C11 cells never reached a plateau, despite a tendency of saturation of the membrane (Fig. 2c). With PrP^null^-1C11 cells, we showed a ~ 25–30% reduction of TiO_2_-NP binding to the plasma membrane whatever the tested concentrations of nanoparticles compared to PrP^C^-expressing 1C11 cells (Fig. 2c), indicating that PrP^C^ takes part in the interaction of TiO_2_-NPs with 1C11 cells.

### TiO_2_ and CB nanoparticles selectively bind full-length PrP^C^

**Fig. 3:**
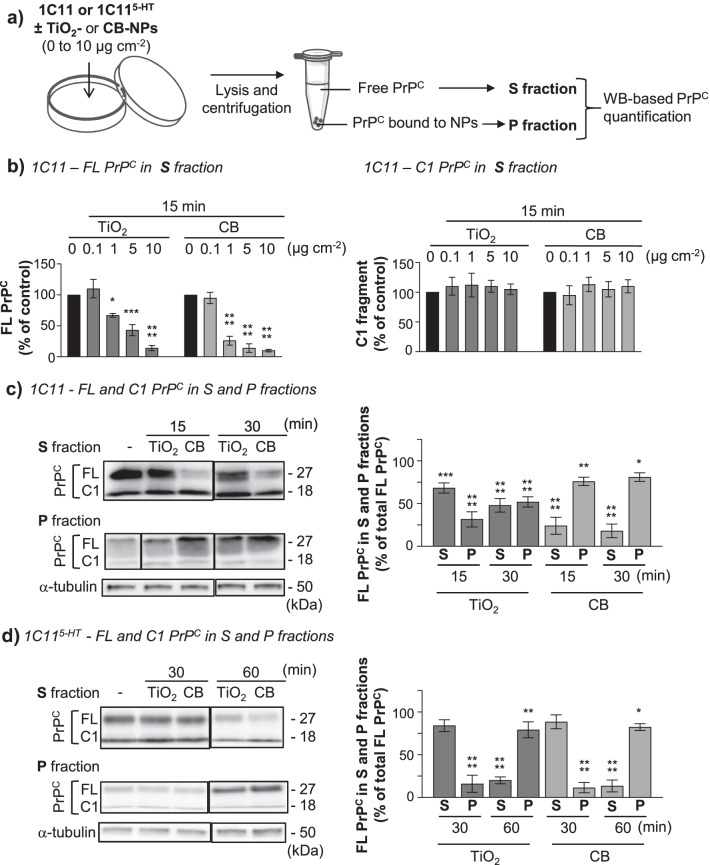
TiO_2_- and CB-NPs specifically interact with full-length PrP^C^ in the 1C11 neuronal cell line. **a** Schematic of the experimental procedure to assess the interaction of TiO_2_- and CB-NPs with PrP^C^ expressed at the plasma membrane of 1C11 precursor cells and their serotonergic 1C11^5−HT^ neuronal progenies by western blotting. **b** Quantification histogram deduced from Western-blot experiments showing the amounts of full-length (FL) PrP^C^ (left) and C1 fragment (right) in the supernatant (S fraction) obtained after lysis of 1C11 cells exposed to increasing concentrations of TiO_2_- or CB-NPs (0 to 10 µg cm^−2^) for 15 min and centrifugation of the lysates. **c**, **d** Representative Western-blots and quantification histograms showing time-variation in the level of FL PrP^C^ and C1 fragment in the S and P fractions derived from 1C11 **c** and 1C11^5−HT^ **d** cells exposed to TiO_2_- or CB-NPs (1 µg cm^−2^). α-tubulin was used for normalization of PrP^C^ level in the S fraction. The experiments were performed three times in triplicates. Values are means ± SEM. *denotes *p* < 0.05, **p < 0.01, ***p < 0.001, ****p < 0.0001 versus unexposed cells

At the plasma membrane coexist several isoforms of PrP^C^, that is, full-length PrP^C^ and truncated PrP^C^ between residues 111/112 (also called PrP^C^ C1 fragment). Full-length and truncated PrP^C^ can be non, mono-, or bi-glycosylated (for review, see [52] and references therein). To assess which PrP^C^ isoforms would be targeted by TiO_2_- or CB-NPs, cells were exposed to 0–10 µg cm^−2^ nanoparticles (Additional file 2: Fig. S1) from 15 to 60 min at 37 °C and lyzed. Cell lysates were centrifuged at 21,130 g to separate free soluble PrP^C^ in the supernatant (S fraction) from PrP^C^ trapped by nanoparticles in the pellet (P fraction). Both fractions were assayed for PrP^C^ Western-blotting after PNGase treatment to remove glycosylations (Fig. 3a).

With 1C11 precursor cells, we observed depletion of full-length (FL) PrP^C^ in the S fraction at 15 min exposure that started at 1 µg cm^−2^ TiO_2_- or CB-NPs (Fig. 3b, left). The decrease in FL PrP^C^ level in the S fraction depended on the concentration of nanoparticles: higher was the concentration of TiO_2_- and CB-NPs, more pronounced was the reduction of FL PrP^C^ amount (Fig. 3b, left). With 10 µg cm^−2^ TiO_2_- or CB-NPs, FL PrP^C^ was quite undetectable in the S fraction after 15 min exposure to nanoparticles (Fig. 3b, left). By contrast, the level of the C1 fragment kept constant whatever the concentration of NPs (Fig. 3b, right). This indicates TiO_2_- and CB-NPs interact specifically with full-length PrP^C^.

Of note, as the depletion of plasma membrane FL PrP^C^ induced by TiO_2_- and CB-NPs was very strong at 5 µg cm^−2^ and upper nanoparticle concentrations, it may compromise the identification of PrP^C^-coupled signaling pathways dysregulated by nanoparticles. For the next experiments, we decided to fix the used concentration of TiO_2_- and CB-NPs to 1 µg cm^−2^. In these conditions, the depletion of FL-PrP^C^ induced by the exposure of 1C11 cells to TiO_2_-NPs (1 µg cm^−2^) at 15 and 30 min was less pronounced than that observed with CB-NPs (Fig. 3c). Reciprocally, the amount of FL PrP^C^ accumulated in the P fraction was more important when 1C11 cells were exposed to CB-NPs than to TiO_2_-NPs (Fig. 3c). This indicates that PrP^C^ present at the plasma membrane of 1C11 cells displays a higher affinity for CB- than for TiO_2_-NPs, in line with the differential K_D_ values measured with recombinant PrP^C^ (Fig. 1b).

With 1C11^5−HT^ neuronal cells exposed to 1 µg cm^−2^ TiO_2_- or CB-NPs, we also showed in the S fraction specific depletion of FL PrP^C^ and invariance in the level of the C1 fragment (Fig. 3d). Conversely, FL PrP^C^ was found to accumulate in the P fraction, while the C1 fragment was quite undetectable (Fig. 3d). As compared to 1C11 precursor cells (Fig. 3c), we observed a time-delay in nanoparticle interaction with FL PrP^C^ expressed by 1C11^5−HT^ neuronal cells: FL PrP^C^ depletion in the S fraction and reciprocal accumulation of FL PrP^C^ in the P fraction (Fig. 3d) started between 30 and 60 min exposure to TiO_2_- or CB-NPs *vs.* 15 min with 1C11 precursors (Fig. 3c). Such difference between 1C11 progenitors and 1C11^5−HT^ neuronal cells likely reflects some neurospecificity of full-length PrP^C^ that could be at the proximal level of cell regionalization (soma *vs.* neurites) and plasma membrane sub-localization (rafts *vs.* non-rafts), glycosylations, and/or the interactome of PrP^C^ [34, 52].

Taken together, these data support the notion that full-length PrP^C^ expressed by 1C11 precursor cells and their serotonergic neuronal progenies is a candidate receptor for TiO_2_- and CB-NPs.

### Interaction of TiO_2_ and CB nanoparticles with PrP^C^ promotes NADPH oxidase-dependent reactive oxygen species production in the 1C11 cell line

Because nanoparticle exposure elicits oxidative stress conditions in epithelial cells [17] and triggers oxidative lesions in the brain of mice [28, 30], we examined whether nanoparticle interaction with full-length PrP^C^ would disturb the PrP^C^-NADPH oxidase functional relationship [38] and promote ROS accumulation.

**Fig. 4:**
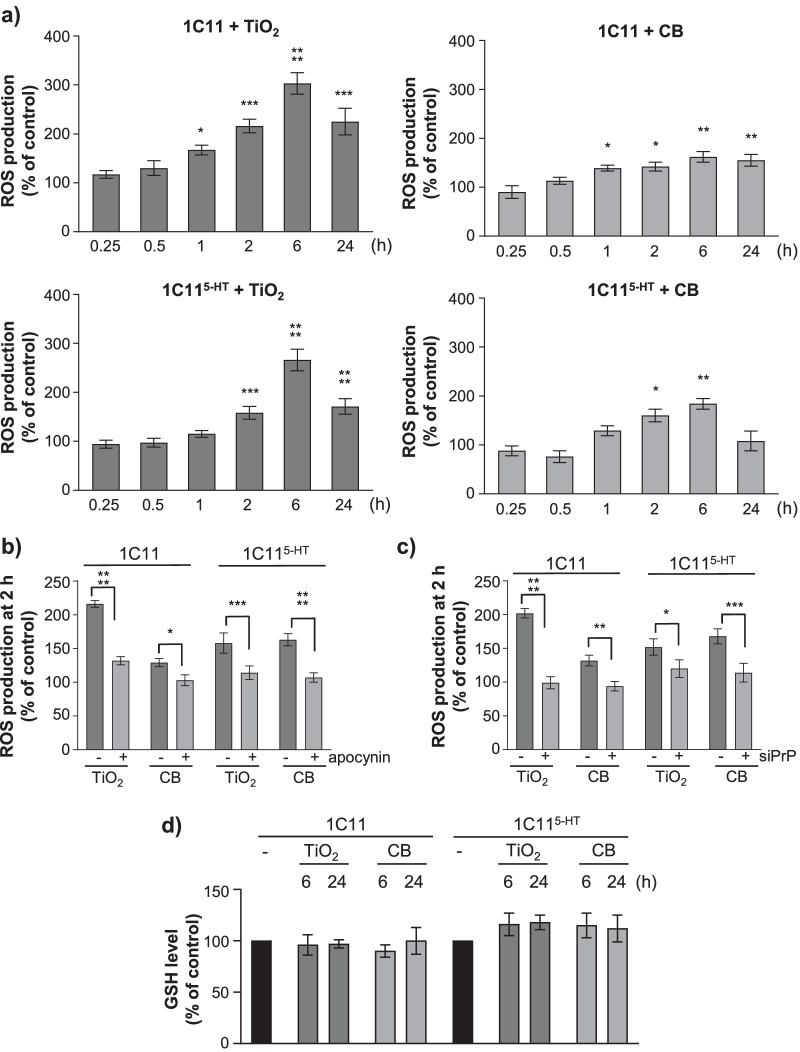
TiO_2_ and CB nanoparticle interaction with PrP^C^ promotes NADPH oxidase-dependent ROS production. **a** Kinetics of ROS production induced on exposure to TiO_2_- and CB-NPs (1 µg cm^−2^) in 1C11 and 1C11^5−HT^ neuronal cells. **b** Involvement of NADPH oxidase in NP-induced ROS production (1 µg cm^−2^ for 2 h) using the NADPH oxidase inhibitor apocynin (500 µM). **c** Quantification histogram showing that siRNA-based PrP^C^ silencing (siPrP) abrogates ROS production induced by TiO_2_- or CB-NPs (1 µg cm^−2^ for 2 h) in 1C11 and 1C11^5−HT^ cells. **d** GSH level keeps constant in 1C11 and 1C11^5−HT^ neuronal cells exposed to TiO_2_- or CB-NPs (1 µg cm^−2^) up to 24 h. The experiments were performed three times in triplicates. Values are means ± SEM. *denotes *p* < 0.05, **p < 0.01, ***p < 0.001, ****p < 0.0001 versus unexposed cells

1C11 precursor cells and their serotonergic 1C11^5−HT^ neuronal progenies were exposed to 1 µg cm^−2^ TiO_2_- or CB-NPs for 15 min to 24 h at 37 °C. The cellular ROS level was measured with the fluorogenic probe CM-H_2_DCFDA that detects both superoxide anions and hydrogen peroxide. Exposure of 1C11 or 1C11^5−HT^ cells to TiO_2_- or CB-NPs triggered ROS production that varied in a time-dependent manner (Fig. 4a). Whatever the nanoparticle type, ROS production started after 1 h exposure in 1C11 cells and 2 h in 1C11^5−HT^ neuronal cells and peaked at 6 h in both cell types. Independently from the cell differentiation stage, the maximal ROS response induced by TiO_2_-NPs was 2.5- to threefold above the basal level. With CB-NPs, the intensity of ROS production was less important than with TiO_2_-NPs with a maximal ROS response 1.5- to 1.8-fold above the basal level in 1C11 and 1C11^5−HT^ cells, respectively.

For both TiO_2_ and CB nanoparticles (1 µg cm^−2^), the induced ROS production was quenched in 1C11 and 1C11^5−HT^ cells when NADPH oxidase was inhibited using the pharmacological inhibitor apocynin (500 µM) (Fig. 4b), indicating that NP-induced ROS production depends on the recruitment of NADPH oxidase. We also monitored that NP-induced ROS generation was canceled in 1C11 cells silenced for PrP^C^ expression (Fig. 4c), supporting corruption of PrP^C^ coupling to NADPH oxidase by TiO_2_- and CB-NPs.

Finally, we probed whether the elevation of ROS level induced by nanoparticles in 1C11 and 1C11^5−HT^ cells would be associated with the onset of oxidative stress conditions by measuring the level of reduced glutathione (GSH) using the CellTracker Green CMFDA fluoroprobe. Whatever the time of exposure (6 or 24 h), treatment of 1C11 or 1C11^5−HT^ cells with TiO_2_- or CB-NPs (1 µg cm^−2^) did not impact the GSH level (Fig. 4d), indicating that such conditions of exposure to TiO_2_- or CB-NPs do not promote oxidative stress in 1C11 precursor cells and their serotonergic neuronal derivatives.

As a whole, these data show that acute exposure to TiO_2_- and CB-NPs provokes PrP^C^-dependent activation of NADPH oxidase and transient ROS accumulation, which modifies the cell redox status without eliciting oxidative stress conditions in the 1C11 cell line.

### Interaction of TiO_2_ and CB nanoparticles with PrP^C^ renders 1C11 and 1C11^5−HT^ cells highly sensitive to pro-inflammatory TNFα cytokine through neutralization of TACE α-secretase activity

We next assessed whether TiO_2_- and CB-NPs would also interfere with the PrP^C^-PDK1-TACE signaling pathway [9, 53]. Dysregulation of this pathway by pathogenic prions PrP^Sc^ or Aβ peptides down-regulates TACE shedding activity, thus leading to the accumulation of type 1 TNFα receptors (TNFR1) at the plasma membrane and thereby rendering prion-infected and Alzheimer’s neurons highly sensitive to TNFα toxicity [9, 41].

**Fig. 5:**
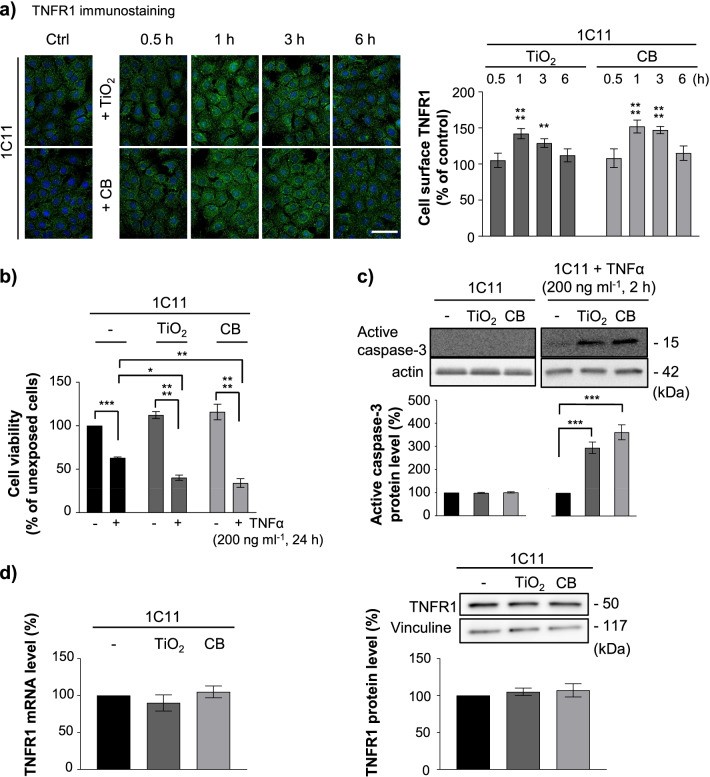
TiO_2_ and CB nanoparticle interaction with PrP^C^ renders 1C11 and 1C11^5−HT^ neuronal cells highly sensitive to TNFα insult by promoting TNFR1 accumulation at the plasma membrane. **a** Kinetics of TNFR1 rise at the plasma membrane of 1C11 cells exposed to TiO_2_- or CB-NPs (1 µg cm^−2^) and related quantification histogram of immunostained TNFR1. Scale bar = 10 µm. **b** Viability and **c** caspase-3 activation in 1C11 cells exposed to TiO_2_- or CB-NPs (1 µg cm^−2^) in combination or not with TNFα (200 ng ml^−1^). **d** TNFR1 expression level as assessed by RT-qPCR and Western-blotting in 1C11 cells exposed to TiO_2_- or CB-NPs (1 µg cm^−2^) for 1 h. Vinculine was used for normalization. The experiments were performed three times in triplicates. Values are means ± SEM. *denotes *p* < 0.05, **p < 0.01, ***p < 0.001, ****p < 0.0001 versus unexposed cells

**Fig. 6:**
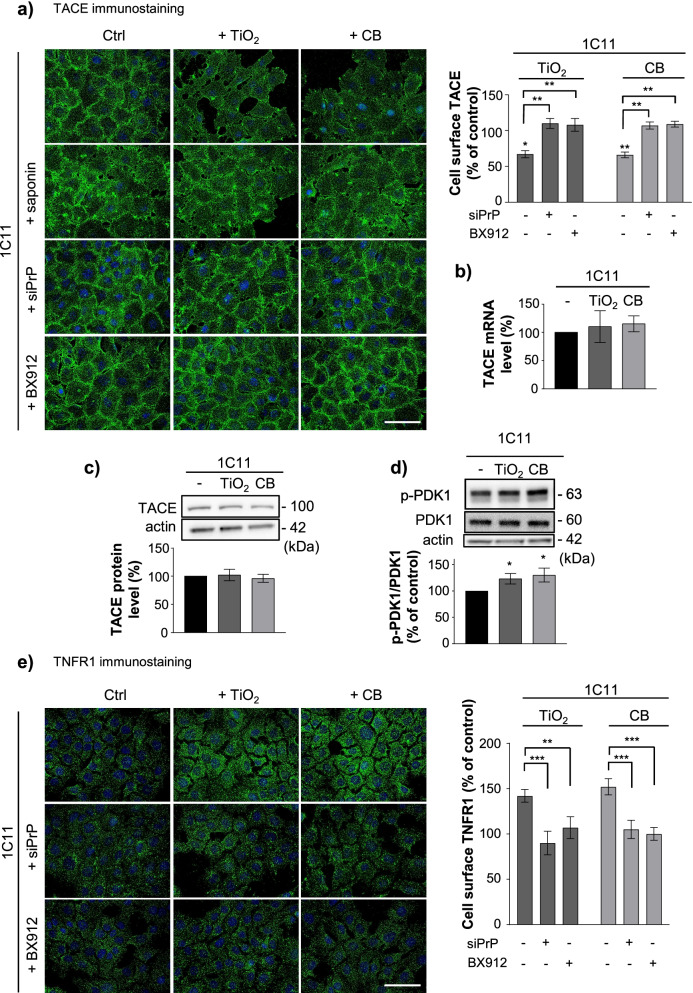
TiO_2_ and CB nanoparticle interaction with PrP^C^ promotes TACE internalization in a PDK1-dependent manner at the root of TNFR1 overexposure at the cell surface. **a** TACE immunostaining at the plasma membrane of 1C11 cells exposed to TiO_2_- or CB-NPs (1 µg cm^−2^) for 1 h in the presence or not of a siRNA toward PrP^C^ (siPrP) or the PDK1 inhibitor, BX912 (1 µM) and related quantification histogram. Cell permeabilization with saponin (0.05%) shows TACE internalization in 1C11 cells exposed to nanoparticles. Scale bar = 10 µm. **b**, **c** TACE expression level as assessed by RT-qPCR **b** and Western-blotting **c** in 1C11 cells exposed to TiO_2_- or CB-NPs (1 µg cm^−2^) for 1 h. **d** PDK1 phosphorylation status at Ser241 (p-PDK1) was assessed by Western-blotting in 1C11 cells exposed to TiO_2_- or CB-NPs (1 µg cm^−2^) for 1 h. **e** TNFR1 immunostaining at the plasma membrane of 1C11 cells exposed for 1 h to TiO_2_- or CB-NPs (1 µg cm^−2^) in the presence or not of a siRNA toward PrP^C^ (siPrP) or the PDK1 inhibitor, BX912 (1 µM) and related quantification histogram. Scale bar = 10 µm. The experiments were performed three times in triplicates. Values are means ± SEM. *denotes *p* < 0.05, **p < 0.01, ***p < 0.001 versus unexposed cells

Acute exposure of 1C11 precursors to TiO_2_- or CB-NPs (1 µg cm^−2^ for 30 min to 6 h) promoted an increase of TNFR1 immunostaining at the plasma membrane as soon as 1 h. The rise in cell surface TNFR1 level was maximal (~ 50% augmentation *vs.* unexposed cells) at 1 h of treatment and maintained until 3 h. TNFR1 level returned to the basal level at 6 h exposure to nanoparticles (Fig. 5a). With serotonergic 1C11^5−HT^ neuronal cells exposed to 1 µg cm^−2^ TiO_2_- or CB-NPs, a maximal augmentation of plasma membrane TNFR1 level was recorded at 4 h (Additional file 3: Fig. S2a). The increase of cell surface TNFR1 did not affect by itself the viability of cells exposed to TiO_2_- or CB-NPs (1 µg cm^−2^) up to 24 h, but rendered NP-exposed cells highly vulnerable to the toxicity of an exogenous insult of TNFα (Fig. 5b), *i.e.*, primed NP-exposed cells to TNFα inflammatory stress. When 1C11 cells were treated for 24 h with TiO_2_- or CB-NPs (1 µg cm^−2^) in combination with TNFα (200 ng ml^−1^), we indeed measured a 60 to 70% reduction of the cell viability, while the sole treatment of 1C11 cells with TNFα promoted a 40% decrease of the viability (Fig. 5b). The increased sensitivity of NP-exposed cells to TNFα was associated with augmented TNFα-mediated caspase-3 activation (Fig. 5c), a downstream effector of TNFR1 death signaling [54]. The overexposure of TNFR1 at the plasma membrane of NP-exposed cells did not result from enhanced TNFR1 gene transcription or translation induced by TiO_2_- or CB-NPs (Fig. 5d) but originated from TNFR1 under-shedding by the α-secretase TACE. Immunofluorescence experiments revealed that the TACE signal was reduced (25–35%) at the surface of cells exposed to TiO_2_- or CB-NPs *vs.* unexposed cells (Fig. 6a, Additional file 3: Fig. S2b).

However, no significant variation in TACE expression was recorded at the mRNA (Fig. 6b) and protein (Fig. 6c) levels between cells exposed or not to nanoparticles. On cell permeabilization with saponin (0.05%), TACE was found intracellularly in those NP-exposed cells (Fig. 6a), indicating NP-induced internalization of TACE.

As for prion-infected and Alzheimer’s neurons [9, 41], we found that TACE internalization in NP-exposed cells and subsequent defect of TNFR1 shedding depended on PrP^C^-mediated activation of the kinase PDK1. TiO_2_- and CB-NPs provoked a rise in PDK1 activity, according to the ~ 1.3-fold increase in the amount of PDK1 molecules phosphorylated on Ser241 in 1C11 cells exposed to nanoparticles (1 µg cm^−2^, 1 h) (Fig. 6d). The siRNA-mediated silencing of PrP^C^ or the inhibition of PDK1 with the pharmacological inhibitor BX912 (1 µM for 30 min prior to NP exposure) prevented the internalization of TACE (Fig. 6a, Additional file 3: Fig. S2b) and plasma membrane accumulation of TNFR1 (Fig. 6e, Additional file 3: Fig. S2a) induced by TiO_2_- or CB-NPs in 1C11 and 1C11^5−HT^ neuronal cells.

Altogether, these data demonstrate that the interaction TiO_2_- or CB-NPs with PrP^C^ corrupts the PrP^C^-PDK1 signaling pathway leading to the internalization of TACE α-secretase. This diverts TACE cleavage activity away from plasma membrane thus leading to cell surface accumulation of TNFR1 at the root of the hypersensitivity of NP-exposed 1C11 and 1C11^5−HT^ cells to TNFα inflammatory stress.

### Corruption of the PrP^C^-PDK1-TACE pathway by TiO_2_ and CB nanoparticles triggers the accumulation of Aβ peptides in 1C11 and 1C11^5−HT^ cells

In prion and Alzheimer’s diseases, we previously showed that PrP^Sc^- or Aβ-induced internalization of TACE α-secretase in neurons also sustains the amyloidogenic processing of APP into neurotoxic Aβ40/42 peptides [9, 42]. We thus sought to determine whether the corruption of the PrP^C^-PDK1-TACE pathway by nanoparticles would trigger a rise in Aβ production.

**Fig. 7:**
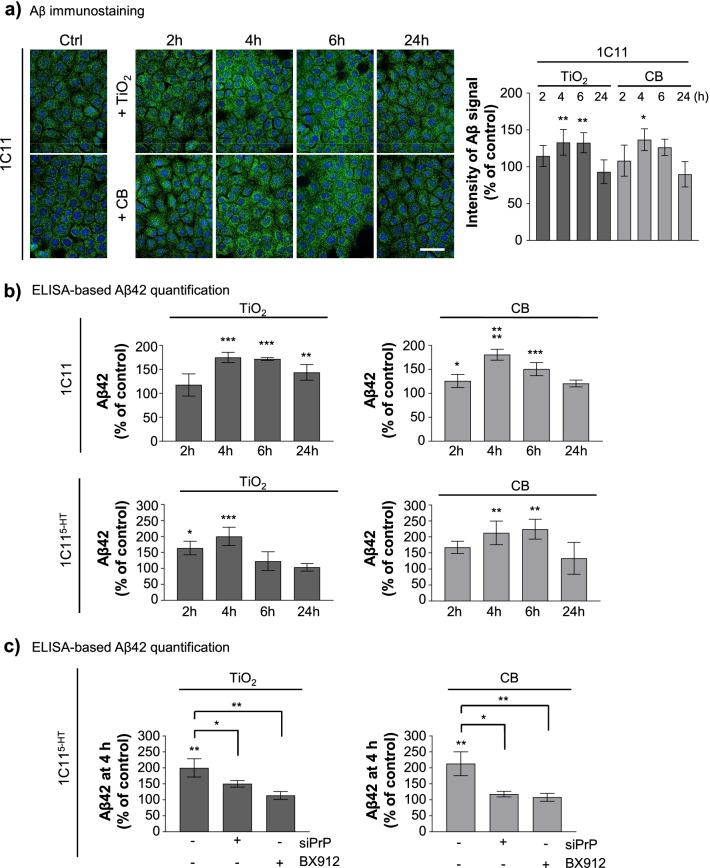
Cell exposure to TiO_2_ and CB nanoparticles promotes rise of Aβ42 through corruption of the PrP^C^-PDK1 pathway. **a** Kinetics of intracellular Aβ accumulation in 1C11 cells exposed to TiO_2_- or CB-NPs (1 µg cm^−2^) and related quantification histogram of immunostained Aβ. Scale bar = 10 µm. **b** ELISA-based quantification of Aβ42 peptides in 1C11 and 1C11^5−HT^ neuronal cells exposed to TiO_2_- or CB-NPs (1 µg cm^−2^) up to 24 h. **c** ELISA-based quantification of Aβ42 peptides in 1C11^5−HT^ neuronal cells exposed to TiO_2_- or CB-NPs (1 µg cm^−2^) for 4 h in the presence or not of a siRNA toward PrP^C^ (siPrP) or the PDK1 inhibitor, BX912 (1 µM). The experiments were performed three times in triplicates. Values are means ± SEM. *denotes *p* < 0.05, **p < 0.01, ***p < 0.001, ****p < 0.0001 versus unexposed cells

Immunofluorescence experiments (Fig. 7a) and ELISA-based quantifications (Fig. 7b, Additional file 4: Fig. S3a) showed that acute exposure of 1C11 and 1C11^5−HT^ cells to TiO_2_- or CB-NPs (1 µg cm^−2^) for 2 to 24 h promoted the transient intracellular rise of Aβ40 and Aβ42 peptides. Accumulation of Aβ40/42 peptides started between 2 and 4 h exposure to nanoparticles in both 1C11 and 1C11^5−HT^ cells and reached a maximum between 4 and 6 h with an Aβ peptides level 1.5- to 1.8-fold above the basal level for 1C11 cells and 2.0- to 2.5-fold above the basal level for neuronal cells. We excluded that the rise of Aβ peptides induced by nanoparticles originated from increased APP and/or β-secretase (BACE1) expression as no significant variation of APP or BACE1 mRNAs and proteins were measured in cells exposed to TiO_2_- or CB-NPs for 4 h *versus* unexposed cells (Additional file 4: Fig. S3b). Of note, the silencing of PrP^C^ or the inhibition of PDK1 with BX912 (1 µM) precluded the accumulation of Aβ40/42 peptides induced by TiO_2_- or CB-NPs (Fig. 7c, Additional file 4: Fig. S3c). This shows the capacity of TiO_2_- and CB-NPs to enhance the production of neurotoxic Aβ peptides of Alzheimer’s disease through their interaction with PrP^C^ and subversion of PrP^C^ coupling to the PDK1-TACE-APP pathway.

### Brain exposure to TiO_2_ nanoparticles triggers TNFR1 accumulation in mice

**Fig. 8:**
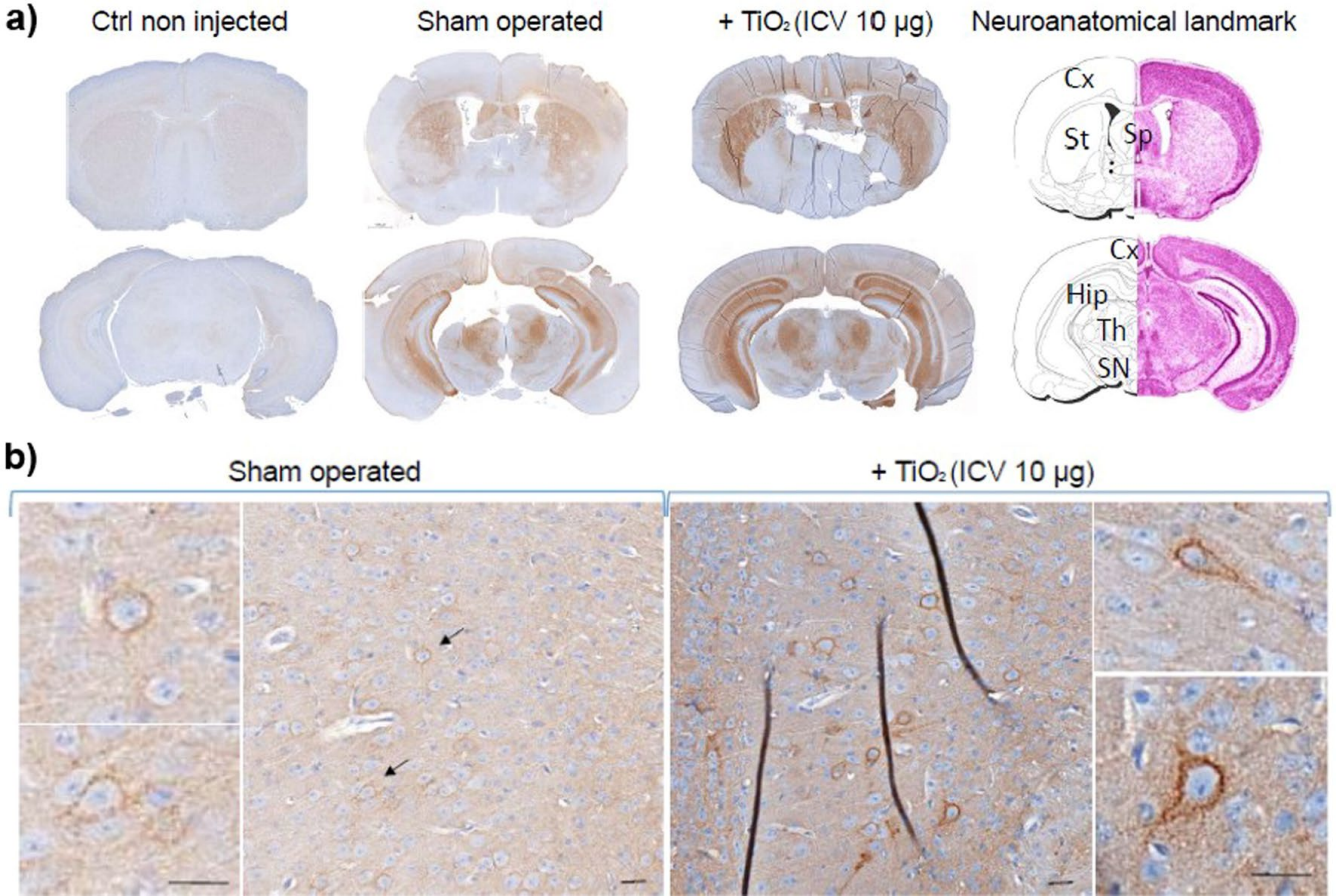
TiO_2_ nanoparticles trigger TNFR1 accumulation in vivo. **a** TNFR1 immunostaining on two representative brain sections from non-injected, sham-operated, and mice exposed to 10 µg of TiO_2_-NPs delivered by an unilateral intra-cerebro-ventricular (ICV) injection 8 weeks before. TNFR1 was detected in the cortex (Cx), septum (Sp) striatum (St), hippocampus (Hip), thalamic (Th) nuclei, and *substantia nigra* (SN). **b** Higher magnification of the subicular cortex shows TNFR1 rise at the plasma membrane of neurons that is stronger in TiO_2_-NPs-injected mice than in sham-operated mice (arrows). Scale bar = 25 µm

To assess the in vivo relevance of our data obtained with the 1C11 neuronal cell line, we exploited mouse brain material derived from a previous proof-of-concept study, in which the brain of female C57Bl/6 J mice was exposed to a single dose of TiO_2_ (P25) nanoparticles (10 µg) via the intra-cerebro-ventricular (ICV) route [55]. As compared to unexposed or sham-operated mice, mice injected with TiO_2_-NPs exhibited a significant, progressive, and severe locomotor deterioration over 8 weeks. These behavioral abnormalities were associated with neuroinflammatory processes, according to microglial activation throughout the brain of TiO_2_-NPs injected mice [55]. We thus sought to assess whether a rise in TNFR1 level, *i.e.*, a cell priming to TNFα toxicity, would accompany the neuroinflammatory state induced by TiO_2_-NPs. Immunohistochemical analyses of TNFR1 on two representative brain sections showed that the basal level of TNFR1 was very faint and roughly not detectable in the brain of non-injected mice (Fig. 8a). In sham-operated mice, a rise of TNFR1 immunostaining occurred in different brain areas, including the cortex, septum, striatum, hippocampus, thalamus, and *substantia nigra*, suggesting a post-surgery effect (Fig. 8a). Mice ICV*-*injected with TiO_2_-NPs displayed a higher TNFR1 level than sham-operated mice in the same above-mentioned brain structures (Fig. 8a), indicating that TiO_2_-NPs promote an augmentation of TNFR1 in vivo. As illustrated in the subicular region of the cortex (Fig. 8b), a higher magnification examination of the brain revealed that the rise of TNFR1 induced by TiO_2_-NPs notably occurred in cells, whose shape and location (grey matter nuclei) are evocative of neurons. As for the 1C11 cell line, the accumulation of TNFR1 in the brain of mice exposed to TiO_2_-NPs would reflect a deficit of TACE shedding activity toward TNFR1. Because NPs stimulate the production of TNFα [55], such an augmentation of TNFR1 in the brain of TiO_2_-NPs injected mice would render neurons hyper-vulnerable to neuroinflammation.

## Discussion

Our work substantiates that TiO_2_- and CB-NPs exert adverse effects on neuronal cells and trigger molecular signs of Alzheimer’s disease through their interaction with non-pathological cellular prion protein PrP^C^ and the corruption of PrP^C^ neuroprotective signaling function. The interaction of TiO_2_- and CB-NPs with plasma membrane PrP^C^ promotes (i) the production of ROS through NADPH oxidase and (ii) the internalization of TACE α-secretase, a cell event that not only primes neurons to TNFα inflammatory stress but also stimulates the amyloidogenic processing of APP, leading to the accumulation of neurotoxic Aβ peptides. Finally, we show that intra-cerebro-ventricular injection of TiO_2_-NPs in C57Bl/6 J mice triggers the accumulation of TNFα receptors in several brain areas that would render the CNS more vulnerable to neuroinflammation.

In detergent-resistant microdomains, *i.e.* lipid-rafts or caveolae, of the plasma membrane, PrP^C^ physiologically behaves as a receptor or co-receptor that takes part in the homeostasis of neuronal and non-neuronal cells [36, 37]. Beyond PrP^C^ capacity to bind pathogenic prions PrP^Sc^ in prion diseases [43], amyloid Aβ peptides in Alzheimer’s disease [33], or α-synuclein in Parkinson’s disease [56], our data show that PrP^C^ can also fix with high-affinity (10 to 30 µg ml^−1^) two types of nanoparticle aggregates, TiO_2_- and CB-NPs, despite distinct size, chemical compositions and reactive properties [47]. This provides prime evidence that the spectrum of molecules recognized by PrP^C^ is not limited to aggregates of misfolded proteins. Of note, individual TiO_2_- and CB-NPs are less than 15 nm but spontaneously aggregate/agglomerate in complex, polydispersed structures in biological media with a mean hydrodynamics diameter of 100–600 nm depending on the concentration of NPs (Additional files 1 and 2: Table S1 and Fig. S1) [17]. Besides, in vitro biochemical studies with Aβ peptides indicate that PrP^C^ binds spherical Aβ oligomers between 6 and 120 nm in diameter that likely corresponds to hexamers or dodecamers of Aβ peptides for the smallest oligomers and an assembly of 5–10 × 10^4^ Aβ molecules for the largest, while monomers of Aβ do not interact with PrP^C^ [57]. It is tempting to speculate that the size and shape of the assembled objects are key parameters for their recognition by PrP^C^.

Which PrP^C^ region(s) interact(s) with TiO_2_- or CB-NPs? For Aβ peptides, three binding domains were identified on full-length PrP^C^, mostly in the N-terminal 23–110 flexible part of PrP^C^ [57, 58]. This PrP^C^ region is composed of octareapeat motifs that bind divalent ions such as copper (amino acids 51–90) [59, 60] and might also attract nanoparticles. Supporting this hypothesis, we show that TiO_2_- and CB-NPs bind full-length PrP^C^ (amino acids 23–230) at the plasma membrane, but do not interact with the C1 fragment of PrP^C^ (amino acids 111–230), that is, the truncated form of PrP^C^ devoid of octarepeat motifs that coexists with full-length PrP^C^ at the cell surface.

As with PrP^Sc^ or Aβ peptides [9, 33, 44, 61, 62], we provide evidence that binding of TiO_2_- or CB-NPs on PrP^C^ at the cell surface of neuronal cells engages and corrupts PrP^C^ signaling function (Additional file 5: Fig. S4). Such nanoparticles/PrP^C^ interaction notably recruits PrP^C^ coupling to the NADPH oxidase/ROS production [38]. Acute exposure of neuronal cells to TiO_2_- or CB-NPs provokes transient activation of NADPH oxidase and subsequent production of ROS. In the 1C11 cell line, the nanoparticle-induced ROS production does not promote oxidative stress conditions, which contrasts with oxidative lesions in the brain of animals exposed to TiO_2_-NPs [28, 30]. These discrepancies may firstly reflect the acute *versus* chronic exposure of neurons to nanoparticles. In animals, repetitive contacts of neurons with nanoparticles likely occur, which in turn might promote sustained activation of NADPH oxidase and generate oxidative stress. Alternatively, the oxidative damages observed in the brain of nanoparticle-treated animals may reflect late events in nanoparticle-induced adverse effects associated with the internalization of TiO_2_- and CB-NPs [63] and their targeting to the lysosomal compartment or NP action on mitochondria [64, 65] at the root of severe ROS production (for review, see [66] and references therein, [17]). In any case, the apparent PrP^C^-dependent nanoparticle-induced non-toxic ROS production we show in neuronal cells would alter the neuronal phenotype by changing the status of PrP^C^-controlled redox-sensitive targets, such as (i) the stress-activated ERK1/2 MAP kinases [38], (ii) the CREB transcription factor [67], or (iii) the serotonin synthesis enzyme Tryptophan Hydroxylase [68], all of them being involved in the fine-tuning of neuronal functions and plasticity.

Most importantly is the dysregulation of the PrP^C^-PDK1-TACE α-secretase pathway [9, 41, 42, 53] by TiO_2_- and CB-NPs (Additional file 5: Fig. S4). First, nanoparticle-induced PDK1 overactivation and subsequent TACE internalization cancel TACE-mediated TNFR1 shedding, thus promoting TNFR1 accumulation at the plasma membrane of neuronal cells and increase of the intrinsic sensitivity of nanoparticle-exposed neurons to exogenous TNFα toxicity. Such an increase of TNFR1 level provoked by nanoparticles was not restricted to cultured neurons, as we monitored in vivo in the brain of mice ICV-injected with nanosized TiO_2_ material the accumulation of TNFR1 at the plasma membrane of groups of neurons that belong to several brain areas, including the hippocampus, cortex, striatum and *substantia nigra*. Associated with the onset of inflammatory stress conditions (for review, see [3] and references therein), the enhanced vulnerability of those nanoparticle-exposed neurons to TNFα would lead to neurodegeneration, possibly accounting for the loss of locomotor abilities [55] as well as the alteration of other brain functions such as memory skills. The in vivo experiment with mice intracerebrally inoculated with TiO_2_ nanoparticles is a proof-of-concept showing that once TiO_2_ nanoparticles have reached the central nervous system they induce adverse effects evocative of Alzheimer’s disease. Additional in vivo experiments are needed to assess whether (i) CB-NPs would also promote TNFR1 rise and sensitize neurons to inflammatory stress in the brain of mice, and (ii) a more realistic exposure to TiO_2_ or CB-NPs notably by the airways would provoke such abnormalities in the brain. Second, the internalization of TACE by both TiO_2_- and CB-NPs diverts TACE α-cleavage neuroprotective activity away from APP. APP thus engages in the β-amyloidogenic processing cascade at the root of the accumulation of neurotoxic Aβ40/42 peptides [69] in 1C11 precursors and serotonergic 1C11^5−HT^ neuronal cells exposed to nanoparticles. Having in mind that PrP^C^ interaction with Aβ peptides relays Aβ toxicity [33, 44] and amplifies Aβ production in AD [9], nanoparticles, by stimulating the production of Aβ40/42 peptides, might start a vicious circle of Aβ production/amplification, thus contributing to the onset of *idiopathic* Alzheimer’s disease.

## Conclusions

The major finding of this in vitro and in vivo study is that the normal cellular prion protein PrP^C^ serves as a binding receptor for aggregates of both TiO_2_ and CB nanoparticles in neurons and transduces NP-associated toxic signals. Despite TiO_2_- and CB-NPs were reported to trigger distinct toxic mechanisms in lung epithelial cells [47], the dysregulation of PrP^C^ signaling function by those two types of NPs emerges as a common mechanism of NP toxicity in neurons. The distortion of PrP^C^-coupled signals by TiO_2_- and CB-NPs not only modifies the redox equilibrium but also renders neurons highly sensitive to TNFα-inflammatory stress and promotes overproduction of neurotoxic Aβ peptides. Such a plausible mechanism of NP-induced neurotoxicity would account for the oxidative stress, inflammatory environment and the deposition of β-sheet rich amyloids in the brain of mice exposed to CB-NPs [20, 70]. Our study hence provides new insight on how human exposure to some nanoparticles may predispose to neurodegenerative diseases and thus brings support to the hypothesis of a possible causal role of some engineered and environmental nanoparticles in the onset of signs of Alzheimer’s disease. Finally, by identifying molecular mechanisms by which TiO_2_- and CB-NPs corrupt signaling cascades in neurons and alter neuronal homeostasis, the present study may be useful for modern toxicology to understand Adverse Outcome Pathways initiated by nanoparticles in the CNS.

## Methods

### Nanoparticles, recombinant PrP^C^ and antibodies

Titanium dioxide (TiO_2_, P25, Sigma-Aldrich, Saint-Quentin Fallavier, France) and carbon black (CB, FW2, Evonik Industries/Degussa, Essen, Germany) were kindly provided by Dr. Sonja Boland (Unit of Functional and Adaptive Biology, Université Paris Cité). The aeroxide TiO_2_ P25 displays 99% purity and is composed of spherical particles, average diameter 22 nm, and mixed crystallinity with 85% anatase and 15% rutile. CB FW2 has a chemical composition of 97–99% of elemental carbon and 1–2% organic carbon and an average diameter of 13 nm. Lyophilized full-length recombinant mouse PrP was purchased from Alicon AG (Zürich, Switzerland) and refolded into PrP^C^ according to the manufacturer’s instructions. Mouse monoclonal Sha31 PrP antibody was purchased from SPI-Bio (Montigny Le Bretonneux, France). Rabbit polyclonal TNFR1 antibody was from NeoBiotech (Nanterre, France). Rabbit polyclonal TACE antibody was from Biovision (San Francisco, CA, USA). Rabbit polyclonal APP antibody was from Novus Biologicals (Littleton, CO, USA). Rabbit monoclonal BACE antibody was from Cell Signaling (Leiden, The Netherlands). Rabbit polyclonal PDK1 and Ser241 phospho-specific PDK1 antibodies were purchased from Cell Signaling (Leiden, The Netherlands) to measure PDK1 level and indirectly PDK1 activity by Western-blot. For normalization, the following antibodies were used: a mouse monoclonal α-tubuline antibody from Proteintech (Rosemount, IL, USA), a mouse monoclonal β-actin antibody from Invitrogen (ThermoFisher Scientific, MA, USA), and a mouse monoclonal vinculin antibody from Merck (Sigma-Aldrich, St. Louis, MO, USA). The monoclonal β-Amyloid (D54D2) XP^®^ Rabbit antibody was from Cell Signaling (Leiden, The Netherlands).

### Characterization of TiO_2_- and CB-NPs by dynamic light scattering (DLS)

The hydrodynamic diameter of aggregates of TiO_2_- and CB-NPs was measured by DLS. Nanoparticles stock suspensions (2 mg ml^−1^) were sonicated with Bioruptor^®^ Plus Sonication System (Diagenode Inc., Denville, NJ, USA) (20 kHz, 320 W) during 3 cycles of 30 s each. Right after the sonication, NPs were diluted as follow: in PBS 22 °C at 5, 20, 40 and 80 µg ml^−1^ and in DMEM/F12 37 °C at 5, 10, 25 and 50 µg ml^−1^ (these latter concentrations corresponded to exposure doses of NPs between 0.1 and 10 µg cm^−2^ in cell-based experiments). NP suspensions were centrifuged for 2 s at 2000 g to remove large aggregates. The supernatants were then analyzed by DLS. Of note, the 5 µg ml^−1^ concentration of NPs in PBS or DMEM/F12 was too low to estimate the hydrodynamic diameter by DLS. For other NPs concentrations, the hydrodynamic diameter was measured with a Vasco Kin™ particle size analyzer (Cordouan Technology, Pessac, France) in combination with the software NanoKin (V2.3.3.0). The following Vasco Kin Particle Size Analyzer parameters were chosen: temperature (22 °C or 37 °C), laser power (between 70 and 80%), acquisition mode (continuous), and analysis mode (Cumulants). The scattering angle was 170°. Each measurement was performed in triplicate (Additional files 1 and 2: Table S1 and Fig. S1).

### In vitro binding experiments between PrP^C^ and nanoparticles

Increasing concentrations of sonicated nanoparticles diluted in PBS (up to 80 µg ml^−1^) were incubated with recombinant PrP^C^ (2 µM) in PBS (100 µl) for 2 h at 4 °C under gentle agitation. Solutions were then centrifuged at 13,523 g for 30 min at 4 °C to pellet nanoparticles and bound PrP^C^. Fluorescence corresponding to free PrP^C^ was measured in the supernatant (λ_exc_ = 280 nm, slit width = 5 nm; λ_em_ = 340 nm, slit width = 10 nm) using a Cary Eclipse fluorometer (Varian Inc., Agilent Technology). Free PrP^C^ was also quantified by Western-blotting.

### Cell culture, neuronal differentiation of 1C11 cells and exposure to nanoparticles

1C11 cells were grown in Dulbecco’s Modified Eagle Medium (DMEM high glucose, GlutaMAX™ supplement, Gibco) supplemented with 10% fetal calf serum (FCS, Biochrom GmBH, Berlin, Germany). On addition of 1 mM dibutyril cyclic AMP (dbcAMP, Sigma-Aldrich, Darmstadt, Germany) and 0.05% CyclohexaneCarboxylicAcid (Sigma-Aldrich, Darmstadt, Germany), almost 100% of 1C11 cells acquire within 4 days a complete serotonergic phenotype (1C11^5−HT^) [35]. Before cells were exposed to nanoparticles, nanoparticle stock solution (2 mg ml^−1^) was sonicated with Bioruptor^®^ Plus Sonication System (Diagenode Inc., Denville, NJ, USA) (20 kHz, 320 W) during 3 cycles of 30 s each, then diluted to the proper concentration in serum-free DMEM/F-12 without phenol red, centrifuged for 2 s at 2000 g, and immediately used. Prepared nanoparticles were applied on cells grown to 90–95% confluency using the dose metric µg cm^−2^, which refers to the concentration of nanoparticles distributed over the surface of the cell culture plate. As done with other cell paradigms [17, 31], and to identify molecular pathways by which NPs promote a loss of cell homeostasis, 1C11 cells and their serotonergic neuronal progenies were acute-exposed to several concentrations of TiO_2_- or CB-NPs ranging from 0.1 to 10 µg cm^−2^.

### Transmission electron microscopy

Cells, grown to ~ 90% confluence on Aclar film (EMS, Hatfield, PA, USA) in a 24-well plate, were rinsed twice in 0.1 M Phosphate Buffer (PB). The preparations were fixed with 1% phosphate-buffered glutaraldehyde (Sigma-Aldrich, Darmstadt, Germany) for 15 min at room temperature (RT) and rinsed twice with PB. After post-fixation with 0.5% osmium tetroxide in PB for 30 min at room temperature, cell preparations were dehydrated with baths of increasing concentrations of ethanol (50, 70, 95, 2*100%) for 8 min each, followed by two 8 min baths of propylene oxide. Preparations underwent half Araldite/half propylene oxide treatment overnight at RT before two Araldite 2 h treatments at RT. After 48 h of polymerization at 60 °C, 70 μm ultrafine sections were cut in ultramicrotome (Leica, Wetzlar, Germany). The contrast agent used was lead citrate. Sections were observed using transmission electron microscope Hitachi H7500 (Tokyo, Japan) equipped with a AMT Hamamatsu numeric camera (Hamamatsu Photonics, Hamamatsu City, Japan).

### Preparation of cell extracts and western blot analyses

Cells were washed in PBS/Ca/Mg and incubated for 30 min at 4 °C in lysis buffer (50 mM Tris–HCl pH 7.4, 150 mM NaCl, 5 mM EDTA, 1% Triton X-100, and cocktails of protease and phosphatase inhibitors [Roche, Basel, Switzerland]). After centrifugation of the lysate (14,000 × g, 30 min), the concentration of the proteins in the supernatant was measured with the bicinchoninic acid method (Pierce, Rockford, IL, USA).

For the PNGase assays, 15 µg protein extracts were incubated with 500 U peptide N-glycosidase F (New England Biolabs, Ipswitch, MA, USA) for 1 h at 37 °C. Ten micrograms of proteins were resolved by SDS/10% PAGE and transferred to nitrocellulose membranes (Amersham, Arlington Heights, IL, USA). Membranes were blocked with 3% non-fat dry milk in PBS 0.1% Tween 20 for 1 h at RT and then incubated overnight at 4 °C with 0.02 µg ml^−1^ Sha31 primary anti-PrP antibody. Bound antibody was revealed by enhanced chemiluminescence detection using a mouse secondary antibody coupled to HRP (GE Healthcare, UK).

### PrP^C^ silencing

We exploited 1C11 precursor cells stably expressing a shRNA towards PrP^C^, for which PrP^C^ expression is repressed by more than 95% (referred to as PrP^null^-1C11 cells) [50, 53]. 1C11 and 1C11^5−HT^ neuronal cells were transiently transfected with a siRNA against PrP (sense sequence 5′-CAGUACAGCAACCAGAACAdTdT-3′) [40] using lipofectamine 2000 reagent following the manufacturer’s instructions (Invitrogen, Carlsbad, CA, USA).

### Flow cytometry

1C11 or PrP^null^-1C11 cells were trypsinized, rinsed with cold PBS, and exposed to nanoparticles for 15 min on ice, with 0.1% sodium azide to prevent internalization of nanoparticles. After centrifugation for 5 min at 1200 g (4 °C), cells were fixed in 3.6% paraformaldehyde-PBS solution for 15 min, and then washed twice with cold PBS. Cells were analyzed using Amnis ImageStream^x^ platform (Amnis, Proteigene, Saint Marcel, France) and Inspire™ system software (Amnis). Camera magnification was 40×. 785 nm excitation laser was at 0.03 mW. The images were acquired with a normal depth of field, providing a cross-sectional image of the cell with a 4 μm depth of focus. A mask representing the whole cell was defined by the bright-field image, and an internal mask was defined by eroding the whole cell mask by 6 pixels (equivalent to 3 μm, as the size of 1 pixel is 0.5 μm) in order to select the signal coming from nanoparticles attached to the cell surface. The results were analyzed by IDEAS software (Amnis). Values of the mean side scatter (SSC) intensity and cell area were calculated for at least 500 cells per sample.

### ROS detection by fluorescence

Productions of ROS in 1C11 precursor cells, 1C11^5−HT^ neuronal cells, and their counterparts silenced for PrP^C^ expression were assessed using the intracellular fluorogenic reagent CM-H_2_DCFDA according to the manufacturer’s instructions (Molecular Probes, Eugene, OR, USA). Following cell exposure to nanoparticles, the fluorescence was recorded (λ_exc_ = 507 nm, slit width = 10 nm; λ_em_ = 528 nm, slit width = 10 nm) using a Cary Eclipse fluorometer (Varian Inc., Agilent Technology).

### Enzyme inhibition

NADPH oxidase activity was inhibited using apocynin (Sigma-Aldrich, Darmstadt, Germany). PDK1 activity was switched-off with BX912 (Axon Medchem BV, Groningen, The Netherlands).

### Fluorescence measurement of intracellular reduced glutathione

The level of GSH was determined using the GSH sensitive probe Celltracker Green CMFDA (Molecular Probes, Eugene, OR, USA). 1C11 and 1C11^5−HT^ cells were exposed to TiO_2_ or CB nanoparticles (1 µg cm^−2^) up to 24 h. The cells were then washed twice with Hanks’ balanced salt solution (HBSS) buffer (Invitrogen, ThermoFisher Scientific, MA, USA) and further incubated for 30 min at 37 °C in HBSS in the presence of 1 µM fluorogenic reagent. HBSS was removed, and the cells were left to reconstitute in DMEM, 10% FCS for 30 min at 37 °C before lysis. Fluorescence intensity of cell lysates was recorded at λ_em_ = 517 nm (slit width = 5 nm) after excitation at λ_exc_ = 492 nm (slit width = 5 nm) using a Cary Eclipse fluorometer (Varian Inc., Agilent Technology). The reference level of intracellular reduced GSH (100%) was obtained using cells unexposed to nanoparticles.

### Immunofluorescence experiments

Immunofluorescent labelings of PrP^C^, TNFR1, TACE, and Aβ were performed using standard protocols. Briefly, for cell surface detection of PrP^C^, TNFR1 and TACE, cells grown on glass coverslips were washed with cold PBS and fixed with 3.6% paraformaldehyde. Cells were incubated for 1 h at room temperature with the primary antibody (0.5 µg ml^−1^) in blocking buffer (PBS enriched with 2% fetal calf serum) and then with Alexa-Fluor 488 or 594-conjugated secondary immunoglobulins (1 µg ml^−1^; Molecular Probes, Eugene, OR, USA). For the intracellular detection of TACE and Aβ, cells fixed with 3.6% formaldehyde were permeabilized with 0.05% saponin (Sigma-Aldrich, Darmstadt, Germany) in PBS for 15 min at room temperature prior to TACE and Aβ immunostaining. Cell preparations were mounted under coverslips with Fluoromount G (Fisher Scientific, Pittsburgh, PA, USA) and analyzed by wide-field indirect immunofluorescence using a Leica DMI6000 B microscope (Wetzlar, Germany). For all images, out-of-focus haze was reduced by digital deconvolution of sets of 16 serial optical sections recorded at 0.3 µm intervals using the Adaptative Blind Deconvolution in the program Autoquant X (Meyer Instruments, Houston, TX, USA). All pixel values in each focal plane were then summed along z-axis to obtain the final image. Deconvoluted images were subjected to image analysis with the AQUA software [71].

### RNA isolation and real-time quantitative RT-PCR analyses

Total RNA was isolated using Trizol reagent according to the manufacturer’s instructions (Life Technologies). The first-strand cDNA synthesis was performed with the Prime Script RT Master Mix kit (Takara Bio). Quantitative real time PCR was performed at 60 °C using SYBR Green kit (ABgene) in the ABI Prism 7900HT device (Applied Biosystems). Primers used are: TNFR1 forward 5′-AGGGCACCTTTACGGCTTCC-3′ and reverse 5′-GGTTCTCCTTACAGCCACACA-3′, TACE forward 5′-CGGAGGAAGCAGGCTCTG-3′ and reverse 5′-GTTTCTAAGTGTGTCGCAGACTG-3′, APP forward 5′-TCGGAAGTGAAGATGGATGC-3′ and reverse 5′-CCTTTGTTCGAACCCACATC-3′, BACE forward 5′-ACCACCAACCTTCGCTTGCCC-3′ and reverse 5′-AAGGGGTCGTGCCTGCTTGC-3′ and as an internal control Rplp0 forward 5′-TACACCTTCCCACTTGCTG-3′ and reverse 5′-TCTGATTCCTCCGACTCTTC-3′.

### Cell viability assays

The viability of ~ 1 × 10^5^ 1C11 or 1C11^5−HT^ cells exposed or not to nanoparticles for 1 h and then exposed to recombinant murine TNFα (Biosource International, Camarillo, CA, USA) was evaluated by cleavage of the tetrazolium salt WST-1 (Roche, Hoffmann-La-Roche Ltd, CH4070, Basel, Switzerland). Activation of caspase-3 upon cell exposure to nanoparticles and/or TNFα was evaluated by Western-blotting using the cleaved caspase-3 (Asp175) antibody (Cell Signaling, Leiden, The Netherlands).

### ELISA measurements of Aβ40/42

The Enzyme Linked ImmunoSorbent Assay (ELISA) measurements were carried out using the Mouse Aβ40 and Aβ42 ELISA Kits (Invitrogen, ThermoFisher Scientific, MA, USA) in accordance with the manufacturer's instructions. The Aβ42 kit has detection range between 3.12 and 200 pg ml^−1^ and the Aβ40 kit between 7.81 and 500 pg ml^−1^. 5 × 10^7^ 1C11 or 1C11^5−HT^ cells were washed in PBS exposed or not to 1 µg cm^−2^ of TiO_2_- or CB-NPs in serum-free DMEM/F-12 medium up to 24 h. After incubation, cells were washed in PBS and lyzed. The lysates were then sonicated (Diagenode Inc., Denville, NJ, USA) 10 cycles of 30 s each and centrifuged (16,000 × g, 30 min). The quantity of Aβ40 and Aβ42 was then measured in the supernatant by ELISA.

### Ethics statements

All animal experiments have been conducted according to the French and European regulations on care and protection of laboratory animals (French Decree 2013–118 of 1 February 2013  and Directive 2010/63/EU of 22 September 2010). Experimental protocols have been evaluated and approved (11–0042) by the Animal Ethics Committee ComEth (National Committee on the Ethics of Animal Experiments (ANSES/ENVA/UPEC)) and the Ministry of national education, higher education and research. The in vivo experiments were performed in the animal facilities at ANSES-Lyon laboratory, which has the relevant approval to carry out animal work (C 69 387 0801) by licensed people working in the animal experiment unit (license numbers AB: 69 387 531). The animals were housed per group in enriched cages in a temperature-controlled room on a 12 h light/dark cycle and received water and food ad libitum.

### In vivo brain exposure to TiO_2_ nanoparticles

Groups of 15 female C57Bl/6J mice (Charles River, L’Arbresle, France), 6-week-old (average weight 19 g), were deeply anesthetized (150 μl of a solution made of 100 μl Ketamin, 50 μl Xylazin diluted in 400 μl water injected intraperitoneally) before a unique acute administration of TiO_2_ NPs (P25) directly within the brain using a stereotaxic device. This way of exposure allows the direct assessment of TiO_2_ nanoparticles neurotoxicity with the benefit of controlling the amount of nanoparticles delivered. Ventricles were chosen as the site of NPs delivery as it brings the advantage to ease the dissemination of NPs to the entire brain in a short time. A TiO_2_-NP solution (5 μg μl^−1^) was prepared from a stock suspension following the standardized dispersion protocol developed in the Nanogenotox Joint Action (www.nanogenotox.eu) established in order to harmonize and standardize the dispersion of nanoparticles for in vitro and in vivo toxicity testing. Briefly, NPs were suspended in sterile-filtered 0.05% w/v BSA-water at a concentration of 2.56 mg ml^−1^ using high energy probe sonication at 4 °C. The hydrodynamic diameter of the suspension was measured by DLS and presented an average mean of 350 nm. Using a 10 µl Hamilton syringe, 2 µl of freshly sonicated TiO_2_ nanoparticles suspensions were stereotaxically injected into the right side of lateral ventricle (stereotaxic coordinates: anteroposterior: − 0,22 mediolateral: + 1 dorsoventral: − 2,25) over a period of 10 min under the control of a motorized microinjection pump. A sham experiment was performed with the same volume (2 μl) of solvent (sterile-filtered 0.05% w/v BSA-water) into the right side of lateral ventricle of a control group of C57Bl/6J mice. None of the mice presented an overt neurological impairment at the time of NPs injection. At 1, 2, 3, 4, and 8 weeks after initial exposure, 3 mice were euthanized by lethal injection of pentobarbital. After exsanguination, brains were removed, fixed in 10% buffered formalin solution and processed for histochemical analysis following classical paraffin embedding. At 8 weeks after exposure, the greatest neurological effects observed were altered locomotor performances combined to neuroinflammation. For the present study, we selected paraffin-embedded sections of mice brain sacrificed at 8 weeks post-exposure.

### TNFR1 immunohistochemistry

To assess the in vivo impact of TiO_2_ NPs on TNFR1 level in the brain, an immunohistochemical analysis was performed on 5 µm thick brain sections of formalin-fixed, paraffin-embedded tissue prepared according to conventional procedures using specific rabbit polyclonal TNFα receptor 1 antibody (abcam, ab19139). Briefly, immunohistochemistry (including deparaffinization, antigenic retrieval, quenching of endogenous peroxidase activity) was performed on an automated immunostainer (Ventana Discovery XT, Roche, Meylan, France) using Omnimap DAB Kit according to the manufacturer’s instructions. Sections were incubated with the rabbit TNFR1 antibody (diluted at 1:1000). An anti rabbit-HRP was applied on sections. Staining appears with the typical brownish color visualized with the DAB chromogenic substrate. The sections were then counterstained with Gill’s hematoxylin. Finally, the brain sections were scanned with panoramic scan II (3D Histech, Budapest, Hungary) at 20X.

### Statistic analyses

Protein and mRNA levels were quantified using NIH ImageJ software (https://imagej.nih.gov/ij/). For experiments with two groups, Student’s t test was used for unpaired samples. For experiments with 3 or more groups, one-way ANOVA test followed by Dunnett's or Tukey's post hoc test were used for independent samples, when comparing the means to control or the means between them, respectively. Error bars on all graphs represent the S.E.M. A *p-value* < 0.05% was considered significant (sample sizes and *p* values are indicated in figure legends).

## Supplementary Information

**Additional file 1: Table S1.** Hydrodynamic diameter (nm) of TiO_2_- and CB-NPs in PBS at 22 °C measured by DLS. Diameter (nm) of aggregates of TiO_2_ and CB nanoparticles (5 up to 80 µg ml^−1^) measured by DLS after NP sonication, dilution in PBS, and centrifugation for 2 sec at 2000 g to remove large aggregates. The hydrodynamic diameter could not be measured for the 5 µg ml^−1^ NP concentration. The experiments were performed in triplicates.

**Additional file 2: Fig. S1.** Hydrodynamic diameter (nm) of TiO_2_- and CB-NPs measured by DLS in DMEM/F12 at 37 °C. Diameter (nm) of aggregates of TiO_2_ and CB nanoparticles was measured by DLS following sonication of NPs, dilution in DMEM/F12, and centrifugation for 2 sec at 2000 g to remove large aggregates. At 10, 25 and 50 µg ml^−1^, TiO_2_-NPs displayed an average diameter of 130 nm (Polydispersity Index-PDI = 0.21), 206 nm (PDI = 0.53), and 375 nm (PDI = 0.22), respectively, and CB-NPs 171 nm (PDI = 0.13), 188 nm (PDI = 0.15), and 327 nm (PDI = 0.52), respectively. The experiments were performed in triplicates.

**Additional file 3: Fig. S2.** TiO_2_ and CB nanoparticles provoke TACE depletion and TNFR1 accumulation at the plasma membrane of serotonergic 1C11^5-HT^ neuronal cells in a PDK1-dependent manner. TNFR1 **a** and TACE **b** immunostaining at the cell surface of 1C11^5-HT^ neuronal cells exposed for 4 h to TiO_2_- or CB-NPs (1 µg cm^−2^) in the presence or not of the PDK1 inhibitor BX912 (1 µM) and related quantification histograms. Representative images of three experiments performed in triplicates are shown. Values are means ± SEM. * denotes *p* < 0.05 and **p < 0.01 versus unexposed cells.

**Additional file 4: Fig. S3.** TiO_2_ and CB nanoparticles enhance Aβ40 production in 1C11 precursors and 1C11^5-HT^ neuronal cells. **a** ELISA-based quantification of Aβ40 peptides in 1C11 and 1C11^5-HT^ neuronal cells exposed to TiO_2_- or CB-NPs (1 µg cm^−2^) up to 24 h. **b** APP and BACE1 expression level as assessed by RT-qPCR and Western-blotting in 1C11 cells exposed to TiO_2_- or CB-NPs (1 µg cm^−2^) for 4 h. **c** ELISA-based quantification of Aβ40 peptides in 1C11^5-HT^ neuronal cells exposed to TiO_2_- or CB-NPs (1 µg cm^−2^) for 4 h in the presence or not of a siRNA toward PrP^C^ (siPrP) or the PDK1 inhibitor, BX912 (1 µM). The experiments were performed three times in triplicates. Values are means ± SEM. * denotes *p* < 0.05, **p < 0.01, ***p < 0.001, ****p < 0.0001 versus unexposed cells.

**Additional file 5: Fig. S4.** Corruption of PrP^C^-coupled signaling pathways by TiO_2_- and CB-NPs in neuronal cells: toward a pro-Alzheimer effect of some TiO_2_ and CB nanoparticles. Cellular prion protein PrP^C^ is a plasma membrane receptor recognized by TiO_2_- and CB-NPs in neuronal cells. The interaction between full-length PrP^C^ and NPs mobilizes PrP^C^-coupled signaling pathways, leading to (i) the activation of NADPH oxidase and the production of ROS, and (ii) the activation of PDK1 that promotes the internalization of TACE α-secretase and thereby down-regulates TACE shedding activity at the root of plasma membrane TNFR1 accumulation and rise in Aβ40/42 production. Such NP interferences with the PrP^C^ signaling network triggers molecular signs of Alzheimer’s disease: modification of cell redox equilibrium, neuronal priming to TNFα inflammatory stress, and accumulation of neurotoxic Aβ40/42 peptides (Image drawn using Servier medical art).

## Data Availability

All data generated or analyzed during this study are included in this published article and its supplementary information files.

## Abbreviations

1C11: Neuroectodermal stem cells
1C11^5^^−^^HT^: Serotonergic neuronal cells
AD: Alzheimer’s disease
APP: Amyloid precursor protein
Aβ: β Amyloid peptide
BACE1: Beta-site APP cleaving enzyme 1
CB: Carbon black
DLS: Dynamic light scattering
NADPH oxidase: Nicotinamide adenine dinucleotide phosphate oxidase
NP(s): Nanoparticle(s) aggregates
PDK1: 3-Phosphoinositide-dependent kinase 1
PNGase: Peptide-N-glycosidase
PrP^C^: Cellular prion protein
ROS: Reactive oxygen species
TACE: Tumor necrosis factor-α-converting enzyme (ADAM17)
TiO_2_: Titanium dioxide
TNFR: Tumor necrosis factor-α receptor
TNFα: Tumor necrosis factor-α
