# Supplementary material for: Titanium dioxide and carbon black nanoparticles disrupt neuronal homeostasis via excessive activation of cellular prion protein signaling
Source: Part Fibre Toxicol. 2022 Jul 15;19:48. doi: 10.1186/s12989-022-00490-x (PMC9284759; doi:10.1186/s12989-022-00490-x)
Supplement: Supplementary file 1 — Additional file 1: Table S1. Hydrodynamic diameter (nm) of TiO2- and CB-NPs in PBS at 22 °C measured by DLS. Diameter (nm) of aggregates of TiO2 and CB nanoparticles (5 up to 80 µg ml−1) measured by DLS after NP sonication, dilution in PBS, and centrifugation for 2 sec at 2000 g to remove large aggregates. The hydrodynamic diameter could not be measured for the 5 µg ml−1 NP concentration. The experiments were performed in triplicates. [file 12989_2022_490_MOESM1_ESM.pdf]

PBS				
Nanoparticle concentration (μg ml^{-1})	TiO_2		CB	
	Hydrodynamic diameter (nm)	PDI**	Hydrodynamic diameter (nm)	PDI**
5	*		*	
20	179.1	0.18	225	0.62
40	342.1	0.89	393	0.91
80	595.8	0.31	543.2	0.83

* Non-determined

** Polydispersity index
