# Supplementary figures and images for: Titanium dioxide and carbon black nanoparticles disrupt neuronal homeostasis via excessive activation of cellular prion protein signaling
Source: Part Fibre Toxicol. 2022 Jul 15;19:48. doi: 10.1186/s12989-022-00490-x (PMC9284759; doi:10.1186/s12989-022-00490-x)

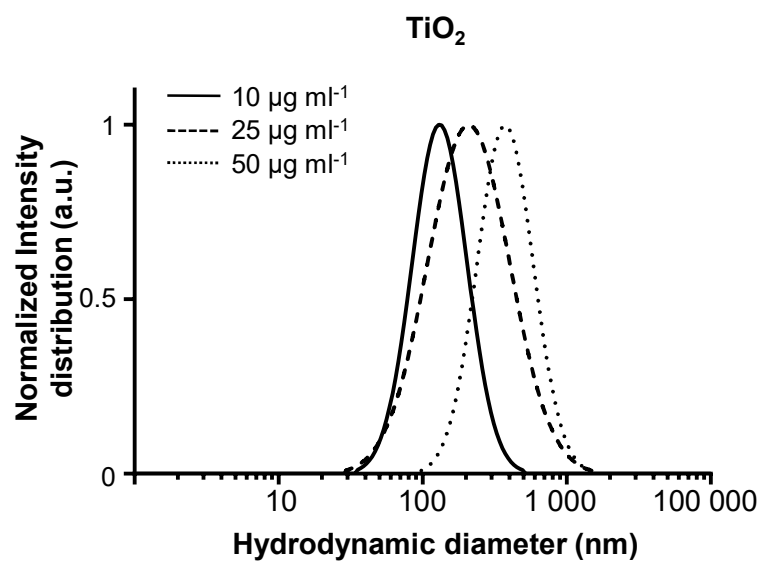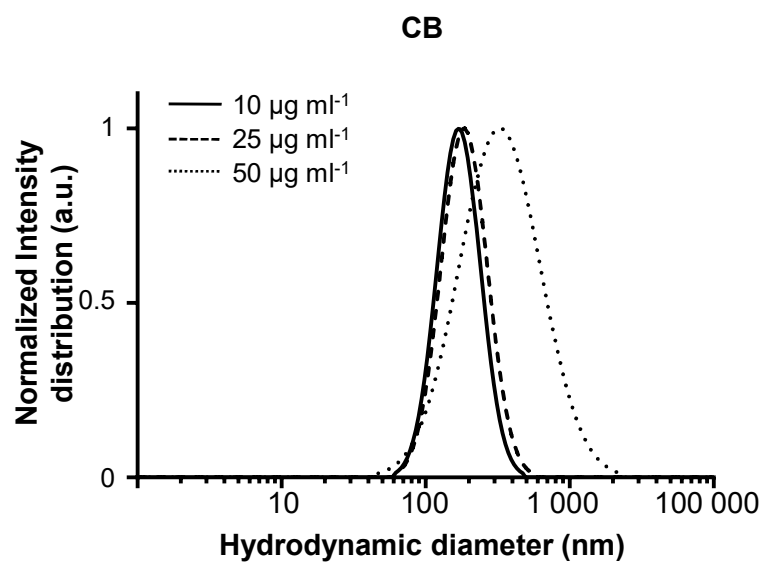

Supplement: Supplementary file 2 — Additional file 2: Fig. S1. Hydrodynamic diameter (nm) of TiO2- and CB-NPs measured by DLS in DMEM/F12 at 37 °C. Diameter (nm) of aggregates of TiO2 and CB nanoparticles was measured by DLS following sonication of NPs, dilution in DMEM/F12, and centrifugation for 2 sec at 2000 g to remove large aggregates. At 10, 25 and 50 µg ml−1, TiO2-NPs displayed an average diameter of 130 nm (Polydispersity Index-PDI = 0.21), 206 nm (PDI = 0.53), and 375 nm (PDI = 0.22), respectively, and CB-NPs 171 nm (PDI = 0.13), 188 nm (PDI = 0.15), and 327 nm (PDI = 0.52), respectively. The experiments were performed in triplicates. [file 12989_2022_490_MOESM2_ESM.pdf]

**a) TNFR1 immunostaining**

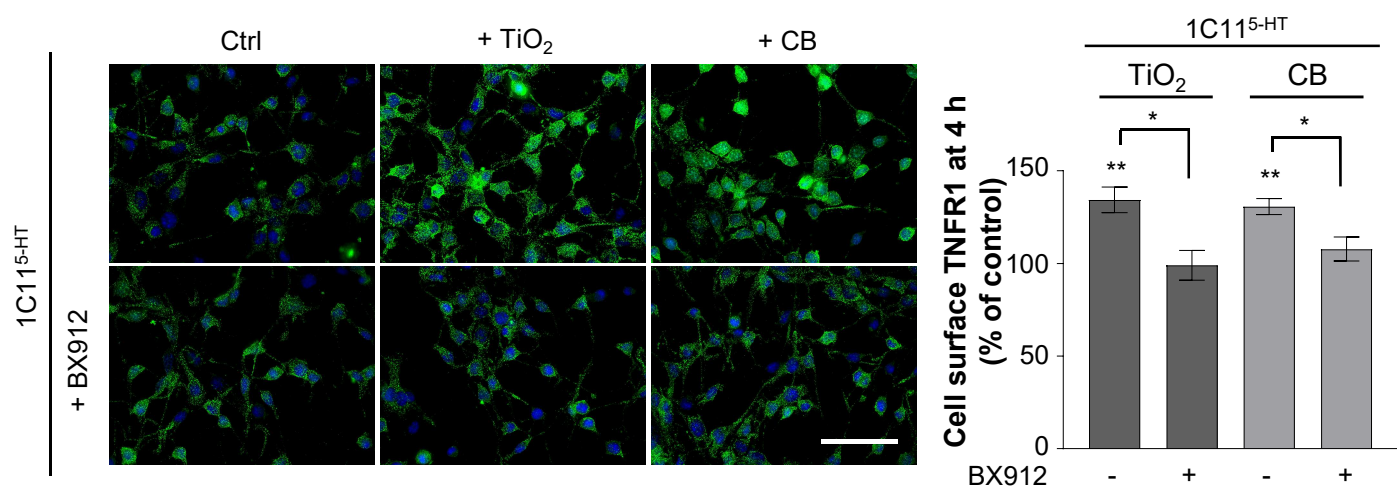

**b) TACE immunostaining**

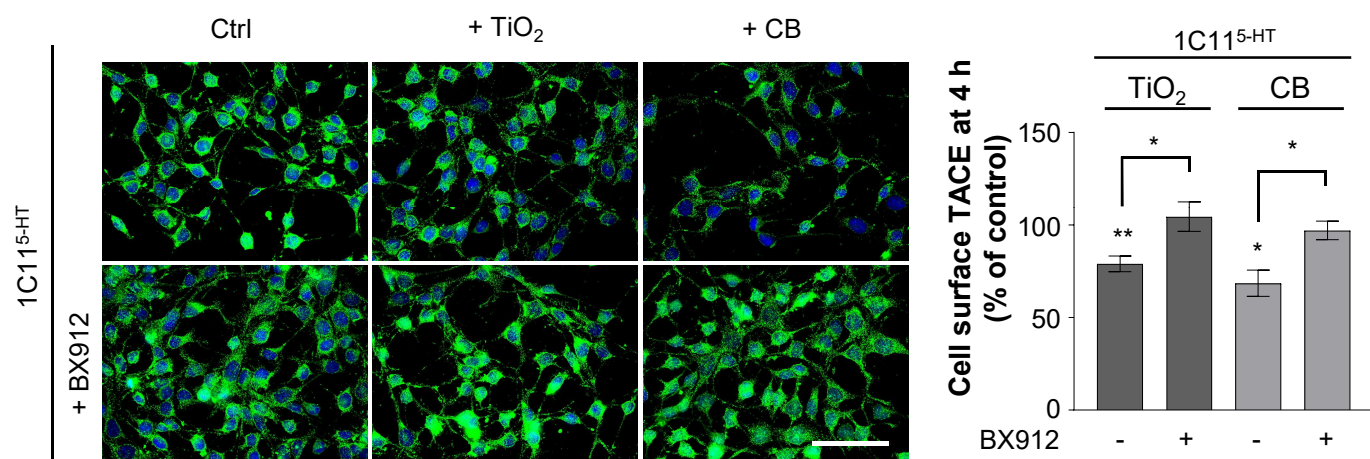

Supplement: Supplementary file 3 — Additional file 3: Fig. S2. TiO2 and CB nanoparticles provoke TACE depletion and TNFR1 accumulation at the plasma membrane of serotonergic 1C115-HT neuronal cells in a PDK1-dependent manner. TNFR1 a and TACE b immunostaining at the cell surface of 1C115-HT neuronal cells exposed for 4 h to TiO2- or CB-NPs (1 µg cm−2) in the presence or not of the PDK1 inhibitor BX912 (1 µM) and related quantification histograms. Representative images of three experiments performed in triplicates are shown. Values are means ± SEM. * denotes p < 0.05 and **p < 0.01 versus unexposed cells. [file 12989_2022_490_MOESM3_ESM.pdf]

**a)** ELISA-based A $\beta$ 40 quantification

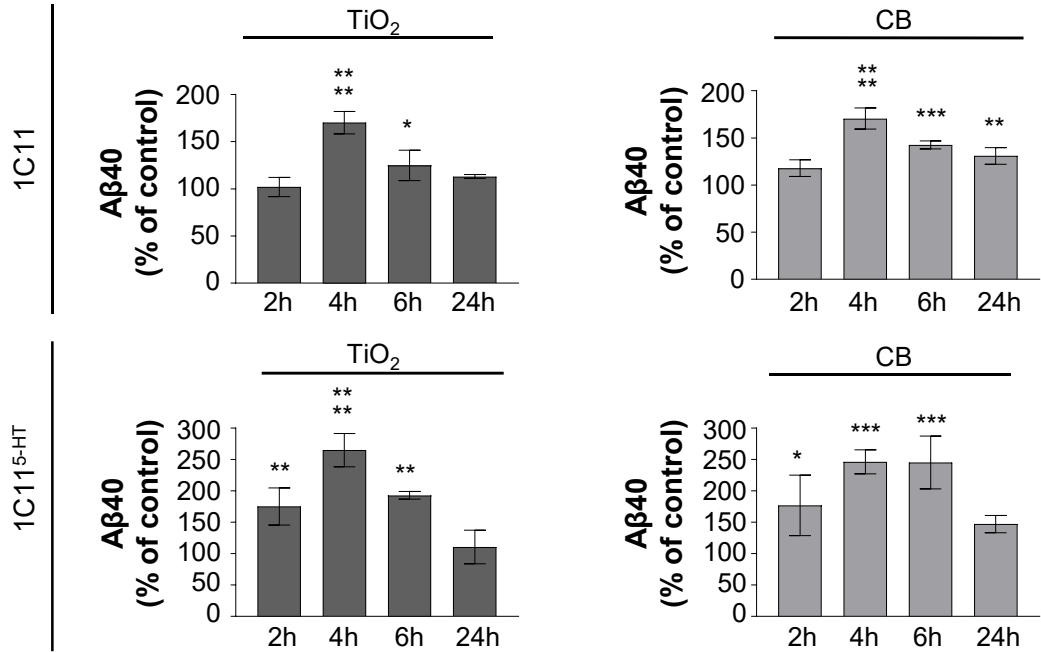

**b)**

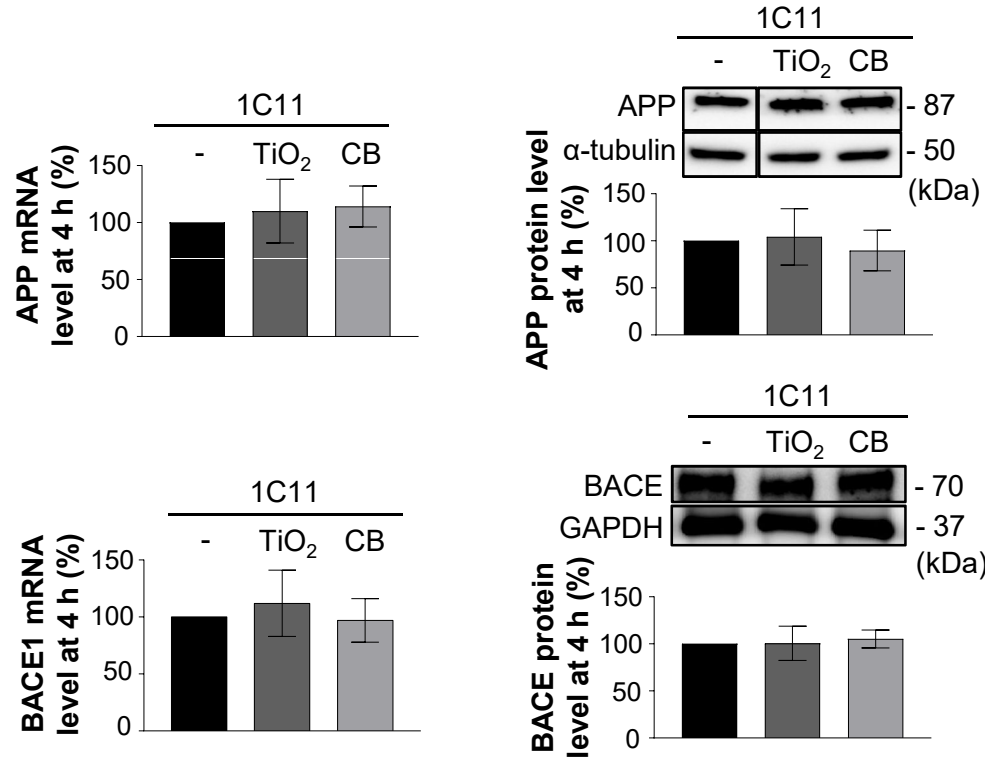

**c)** ELISA-based A $\beta$ 40 quantification

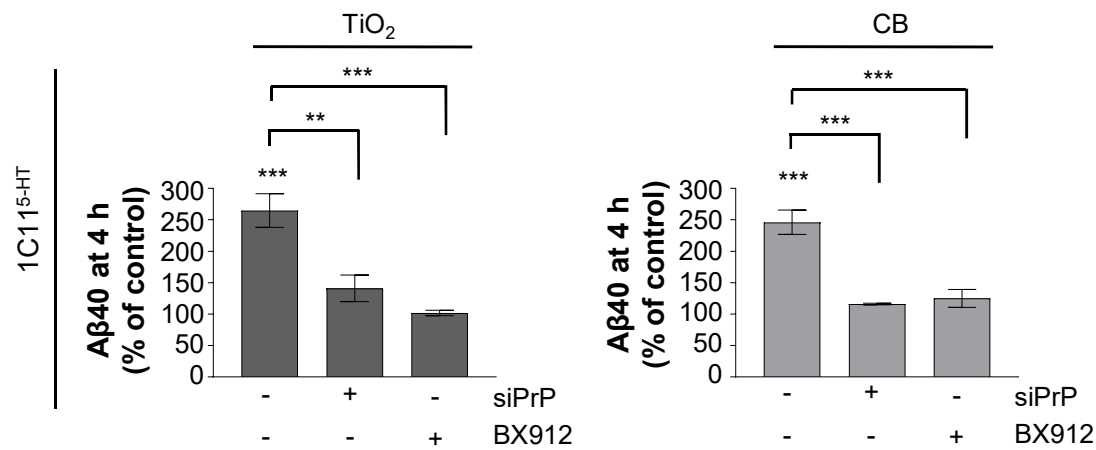

Supplement: Supplementary file 4 — Additional file 4: Fig. S3. TiO2 and CB nanoparticles enhance Aβ40 production in 1C11 precursors and 1C115-HT neuronal cells. a ELISA-based quantification of Aβ40 peptides in 1C11 and 1C115-HT neuronal cells exposed to TiO2- or CB-NPs (1 µg cm−2) up to 24 h. b APP and BACE1 expression level as assessed by RT-qPCR and Western-blotting in 1C11 cells exposed to TiO2- or CB-NPs (1 µg cm−2) for 4 h. c ELISA-based quantification of Aβ40 peptides in 1C115-HT neuronal cells exposed to TiO2- or CB-NPs (1 µg cm−2) for 4 h in the presence or not of a siRNA toward PrPC (siPrP) or the PDK1 inhibitor, BX912 (1 µM). The experiments were performed three times in triplicates. Values are means ± SEM. * denotes p < 0.05, **p < 0.01, ***p < 0.001, ****p < 0.0001 versus unexposed cells. [file 12989_2022_490_MOESM4_ESM.pdf]

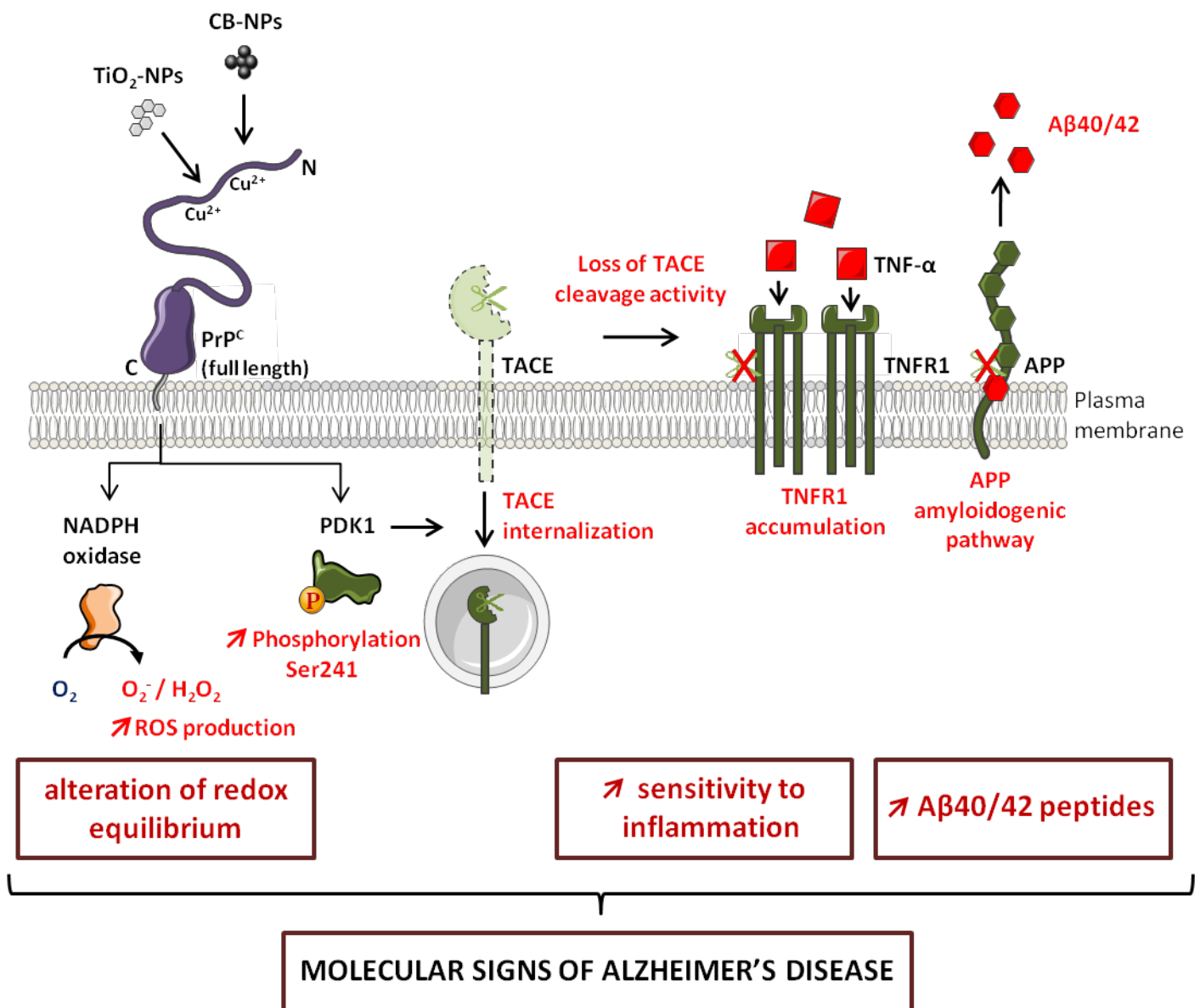

Supplement: Supplementary file 5 — Additional file 5: Fig. S4. Corruption of PrPC-coupled signaling pathways by TiO2- and CB-NPs in neuronal cells: toward a pro-Alzheimer effect of some TiO2 and CB nanoparticles. Cellular prion protein PrPC is a plasma membrane receptor recognized by TiO2- and CB-NPs in neuronal cells. The interaction between full-length PrPC and NPs mobilizes PrPC-coupled signaling pathways, leading to (i) the activation of NADPH oxidase and the production of ROS, and (ii) the activation of PDK1 that promotes the internalization of TACE α-secretase and thereby down-regulates TACE shedding activity at the root of plasma membrane TNFR1 accumulation and rise in Aβ40/42 production. Such NP interferences with the PrPC signaling network triggers molecular signs of Alzheimer’s disease: modification of cell redox equilibrium, neuronal priming to TNFα inflammatory stress, and accumulation of neurotoxic Aβ40/42 peptides (Image drawn using Servier medical art). [file 12989_2022_490_MOESM5_ESM.pdf]
